# COMPAS-4: A Data Set of (BN)_1_ Substituted *Cata*-Condensed Polybenzenoid Hydrocarbons—Data Analysis and Feature Engineering

**DOI:** 10.1021/acs.jcim.5c00608

**Published:** 2025-05-23

**Authors:** Sabyasachi Chakraborty, Itay Almog, Renana Gershoni-Poranne

**Affiliations:** The Schulich Faculty of Chemistry and the Resnick Sustainability Center for Catalysis, 26747 Technion - Israel Institute of Technology, Haifa 32000, Israel

## Abstract

Incorporation of a boron–nitrogen (BN) pair into polycyclic aromatic hydrocarbons is a common approach for modulating their electronic properties. However, a conceptual and quantitative framework rationalizing the observed effects has not been developed, and general structure–property relationships remain elusive. In this work, we perform a data-driven investigation that leads to concrete principles for rational design of (BN)_1_-PBHs with targeted properties. We construct a new chemical database, COMPAS-4, which contains the geometries and properties of all possible (BN)_1_-PBH isomers up to 6 rings, calculated at both the GFN1-xTB and density functional theory (DFT) (CAM-B3LYP/def2-SVP) levels of theory. We investigate the influence of BN-substitution on various molecular properties, including their molecular orbital energies and aromaticity, and define specific structural features that determine these properties. Notably, all of these features are chemically intuitive and simple to extract from the structure of the molecule, without any prior computation. We find that the most influential feature is the number of rings whose cyclic delocalization is disturbed as a result of the substitution.

## Graphical abstract

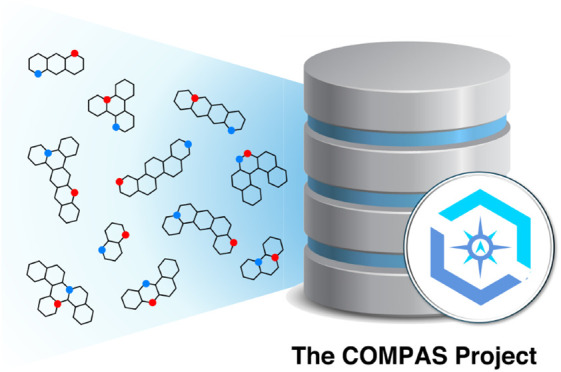

## Introduction

Polycyclic aromatic systems (PASs) are a class of conjugated molecules that are prevalent in many areas of chemistry and materials sciences.^1,2^ They are often considered the workhorse of organic electronics and many examples exist of PASs as organic semiconductors,^3,4^ light-emitting diodes,^5^ field-effect transistors,^6^ organic photovoltaics,^7–9^ and fluorescent emitters.^10,11^ Nevertheless, there is an ongoing search for new functional PASs with tailored properties, which can potentially enable enhanced device performance and new technologies.

One promising direction is embedding boron–nitrogen (BN) pairs into polybenzenoid hydrocarbons (PBHs; PAS comprising only benzene rings) by replacing any two carbon atoms. Though the resulting BN-PBHs are isoelectronic to their respective parent PBHs, the complementary electron-accepting and electron-donating properties of the B and N atoms have a dramatic effect on the (opto)­electronic property space.^12–14^

The earliest attempts to synthesize BN-PBHs were by Dewar and co-workers in the 1960s.^15–17^ For several decades afterward, the field lay dormant, until it recently experienced a renaissance led by Paetzold,^18,19^ Ashe,^20–23^ Piers,^24–27^ Liu groups,^28–31^ and others.^32–41^ These efforts led to the recent identification of functionally important systems—molecular solar thermal systems,^42^ air-stable organo-electronics,^43^ and circularly polarized light emitters.^44^ As experimental interest in these molecules surged, so did computational efforts to characterize and understand the underlying structure–property relationships of BN-PBHs. Yet, this remains a daunting task due to the vastness of the chemical space. The structural diversity stems from three core aspects (Figure 1): the number of rings, the geometry in which the rings are fused, and the placement of the B and N atoms. To provide a sense of scale: enumeration of the chemical space of (BN)_*x*_-substituted PBHs containing *n*_rings_ ≤ 6 (where 1 ≤ *x* ≤ 0.5*n*_C_, *n*_C_ is the number of carbons in the parent PBH, and *n*_rings_ is the number of rings in the molecule) yields 7.4·10^12^ molecules.^45^

**Figure 1:**
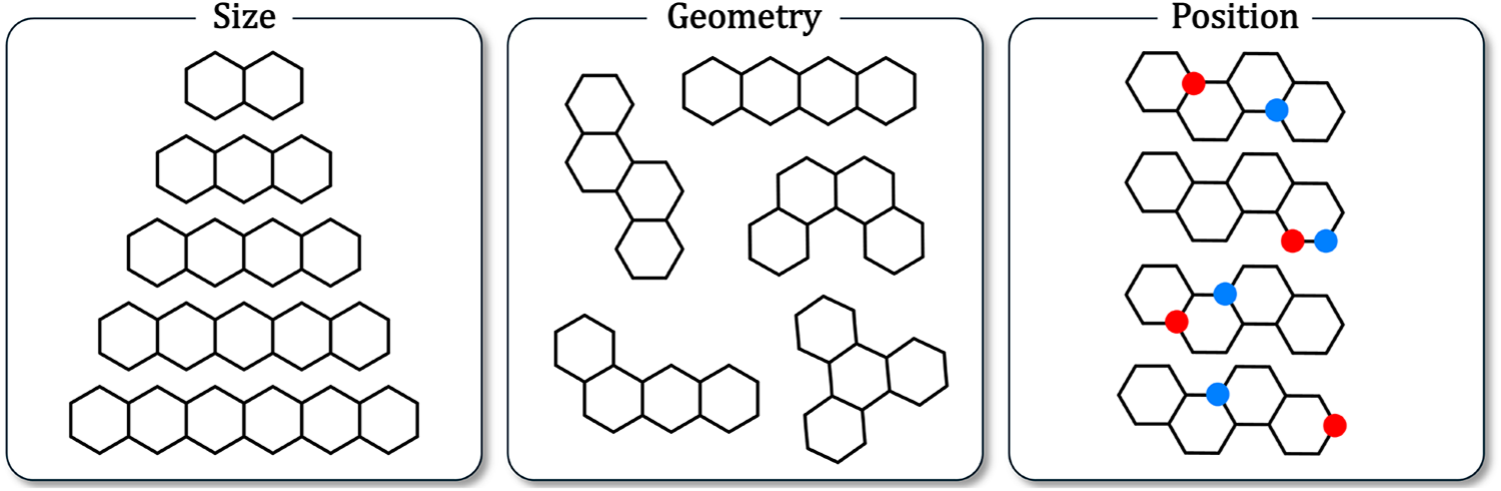
Schematic illustration of the three aspects that provide the structural diversity of (BN)_1_-PBHs. Explicit Hs and double bonds are omitted for clarity. Red: boron, blue: nitrogen.

Both computational and experimental efforts implemented so far have generally focused on studying the positional isomers of a single scaffold. For example, Baranac-Stojanović studied the effect of BN substitution on benzene, naphthalene, and coronene.^46–49^ Similar investigations were carried out into the chemical spaces formed from BN-substitution of benzene,^50^ naphthalene, anthracene,^51^ phenanthrene,^52^ tetracene,^13^ picene,^53^ and perylene.^54^ While such investigations do lead to a deeper understanding of the behavior of various subsets of molecules, they are not conducive to defining general design principles. To do so, it is necessary to explore two aspects concurrently: variations in BN-substitution patterns as well as changes to the structure of the PBH scaffold. Only a comprehensive study of this type may lead to the identification of structure–property relationships that apply to the broad chemical space of BN-PBHs.

In this work, we begin to address this gap by constructing, analyzing, and feature-engineering a new data set, COMPAS-4, which contains all possible *cata*-condensed (cc) (BN)_1_-PBH isomers comprising up to 6 rings. The COMPAS-4 data set contains molecules that differ in their *n*_rings_, their positional BN isomerization, and their cc-PBH scaffold. Investigating this complete and well-defined chemical space ensures the identification of transferable trends. We define a small set of chemically intuitive structural features and demonstrate that these domain-informed features capture many chemical trends, enabling the prediction of various electronic molecular properties, such as the highest occupied molecular orbital–lowest-unoccupied molecular orbital (HOMO–LUMO) gap (Δ*E*_H–L_), without the need for prior quantum chemical calculations.

The newly developed data set is the most recent installment of the COMPAS Project (COMputational database of Polycyclic Aromatic Systems), an open-access database established by our group. The COMPAS database already houses COMPAS-1 (cc-PBHs),^55^ COMPAS-2 (heterocyclic cc-PBHs),^56,57^ and COMPAS-3 (*peri*-condensed PBHs),^58^ which have been used to train both interpretable^59,60^ and generative models.^61^ COMPAS joins other databases, such as NASAs Ames,^62^ PAH335,^63^ FORMED,^64^ OE62,^65^ and OCELOT,^66^ to provide the necessary foundation for data-driven investigations into the important chemical space of PASs.

## Data Generation Workflow

The process of generating the COMPAS-4 data sets involved several steps: enumeration, geometry optimization, data filtration, and curation of desired properties. The following subsections detail these steps.

### Step 1: Structure Enumeration

Our chemical space of (BN)_1_-PBHs was based on the collection of 57 parent scaffolds, i.e., all possible unsubstituted cc-PBHs containing *n*_rings_ ⩽ 6. By replacing any two carbons with a BN pair, a total of 23,894 unique (BN)_1_-PBHs can be generated. All of the (BN)_1_-PBHs included in the data set were isoelectronic to their parent cc-PBH, meaning that the number of hydrogen atoms was not varied, regardless of the position of substitution. The numbers of scaffolds and (BN)_1_-PBH isomers available for each *n*_rings_ are provided in Table 1.

**Table 1:** Numbers of cc-PBHs Scaffolds and (BN)_1_-PBHs Resulting from Different *n*_rings_

*n*_rings_	no. cc-PBH	no. (BN)_1_-PBHs
2	1	23
3	2	137
4	5	741
5	12	3813
6	37	19,180
total	57	23,894

### Step 2: Structure Optimization

The initial Cartesian coordinates of the 23,894 enumerated (BN)_1_-PBHs were extracted from ref 45. The structures were subjected to geometry reoptimization performed with two different computational methods: density functional theory (DFT) and semiempirical. In both cases, the specific level of theory was chosen to maintain consistency and uniformity with the other COMPAS data sets,^55,56,58^ and because it strikes a good balance between accuracy and computational cost. Most importantly, the computed values capture the same trends as the experimental results, showing good agreement and a systematic error that can be straightforwardly corrected. See Section S2 of the Supporting Information for additional benchmarking details.

#### DFT Optimization

DFT optimization was performed with the CAM-B3LYP functional^67^ and the def2-SVP^68^ basis set, using Grimmes D3 dispersion correction^69^ with Becke–Johnson damping,^70,71^ as implemented in Orca 5.0.3 and 5.0.4.^72,73^ We have shown a good agreement between this level of theory and experimental values of *S*_0_–S_1_ ^74^ and *S*_0_–T_1_ ^75^ energy gaps. In Section S2.2 of the Supporting Information, we further show a good agreement between Δ*E*_H–L_ values calculated at this level and experimentally measured absorption wavelengths of a selection of (BN)_1_-PBHs. The resolution-of-identity and the chain-of-spheres approximations (RIJCOSX) were invoked for the Coulomb and exchange integrals, respectively. The AuxJ keyword was used to call the required auxiliary basis sets.^76^ Harmonic vibrational frequencies were calculated at the same level of theory to confirm that all geometries were minima on their respective potential energy surfaces. For each molecule, the respective cation and anion were optimized at the same level of theory including subsequent frequency calculations, as well. Altogether, the geometries of 72k species were optimized.

#### xTB Optimization

The same initial *xyz* coordinates were subjected to optimization with xTB, using GFN1-xTB.^77^ In our previous investigations, we found that GFN1-calculated HOMO and LUMO values for heterocyclic molecules agree much better with DFT than those obtained with GFN2, and slightly better than PM6 and PM7 (see Section S2.1 of the Supporting Information for further details). Following optimization, harmonic vibrational frequencies were calculated to ensure true minima on the potential energy surface. For each molecule, the respective cation and anion were also optimized with GFN1-xTB, including subsequent frequency calculations. Altogether, the geometries of 72k species were optimized.

### Step 3: Data Filtration

We observed that some molecules underwent rearrangements during optimization. In particular, molecules with helical structures tended to undergo undesired bond formation (i.e., cyclization), leading to the wrong structures. These were identified by comparing the InChI descriptors^78^ of the optimized structures (generated using OpenBabel^79^) to the input geometries.

To ensure both data sets contain the same molecules, any molecule that rearranged in any of the six optimizations (two computational methods multiplied by three charge states each) was discarded. Altogether, 38 molecules were removed. The geometries and properties of the remaining 23,856 molecules calculated with GFN1-xTB and with CAM-B3LYP/def2-SVP comprise the COMPAS-4x and the COMPAS-4D data sets, respectively.

All neutral species were found to be minima. However, 1574 DFT-optimized structures (COMPAS-4D) showed imaginary frequencies in the cationic or anionic states (or both). These molecules are included in the data sets but are not considered in the analysis of the aIP and aEA (see the Supporting Information). The data files include a notation identifying these structures. Hence, all analyses on COMPAS-4D data included 22,282 data points.

### Step 4: Property Curation

The properties contained in each of the two data sets are detailed in Table 2, where HOMO and LUMO are the highest occupied and lowest unoccupied molecular orbitals, respectively; Δ*E*_H–L_ is the HOMO–LUMO energy gap; SPE is the dispersion-corrected single-point energy (i.e., the energy of the optimized structure without zero-point corrections); *E*_rel_ is the relative SPE (vide infra); aIP is the adiabatic ionization potential; and aEA is the adiabatic electron affinity. The Gibbs free energy and enthalpy were calculated at 273.15 K.

**Table 2:**
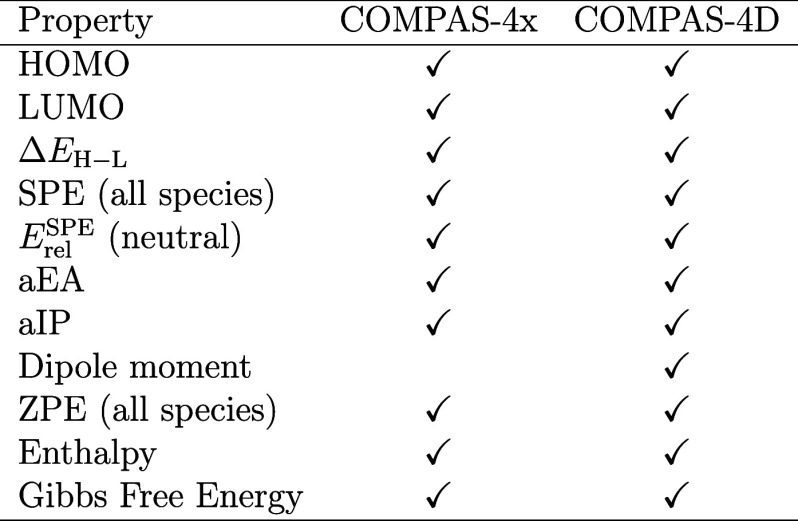
Molecular Properties Included in the COMPAS-4 Data sets

*E*_rel_ (calculated only for the neutral species) was obtained by calculating the SPE difference between each molecule and the most stable molecule of the same size (i.e., the same *n*_rings_). Accordingly, for every *n*_rings_, the lowest value is zero, with all molecules belonging to that subset exhibiting positive *E*_rel_ with respect to the reference isomer.

For a detailed account of the structural and property range distributions for each of the data sets, see Section S4 in the Supporting Information.

## Results and Discussion

This section contains three subsections: domain-informed feature design, structure property relationships, and predictive performance of our feature set. All results in this section are based on the COMPAS-4D data set.

### Domain-Informed Feature Design

In recent years, our group has developed new chemical representations tailored for PASs by identifying which structural features are most dominant in determining molecular properties. Although many other types of representations exist—from very simple (e.g., molecular formula) to complex (e.g., physics-based methods such as Coulomb Matrix,^80^ SOAP,^81^ SLATM,^82^ FCHL,^83,84^ and MAOC)^85^—one of the defining characteristics of our representations is that they are based solely on the connectivity of the molecular structure. As a result, they can be extracted simply by visual inspection of a structure and do not require any quantum-chemical calculations. We have demonstrated that our representations allow faster and more efficient training and, more importantly, that they are interpretable.^59,60^ Chemical insight can be easily extracted from models trained on these representations because they are based on intuitive and clear structural features.

Continuing in the same vein, we sought to identify intuitive and easily extracted structural features that determine the molecular properties of (BN)_1_-PBHs. To account for the polycyclic scaffold as well as the positional isomerism of the B and N atoms, we defined five features of two types: two scaffold-dependent (i.e., based on the PBH parent structure) and three BN-location dependent (Figure 2). The five features are

1. *n*_rings_—the number of rings in the molecule (Figure 2A).
2. *n*_LL_—the length of the longest linear stretch (Figure 2B).
3. B_*i*_/B_*o*_, N_*i*_/N_*o*_—a binary classification for each heteroatom that indicates whether it is located on a fused bond or not (Figure 2C).
4. *n*_SP_—the number of carbons situated between the B and N atoms along the Shortest Path that connects them (Figure 2D).
5. *n*_DR_—the number of Disrupted Rings in the (BN)_1_-PBH (Figure 2E).

**Figure 2:**
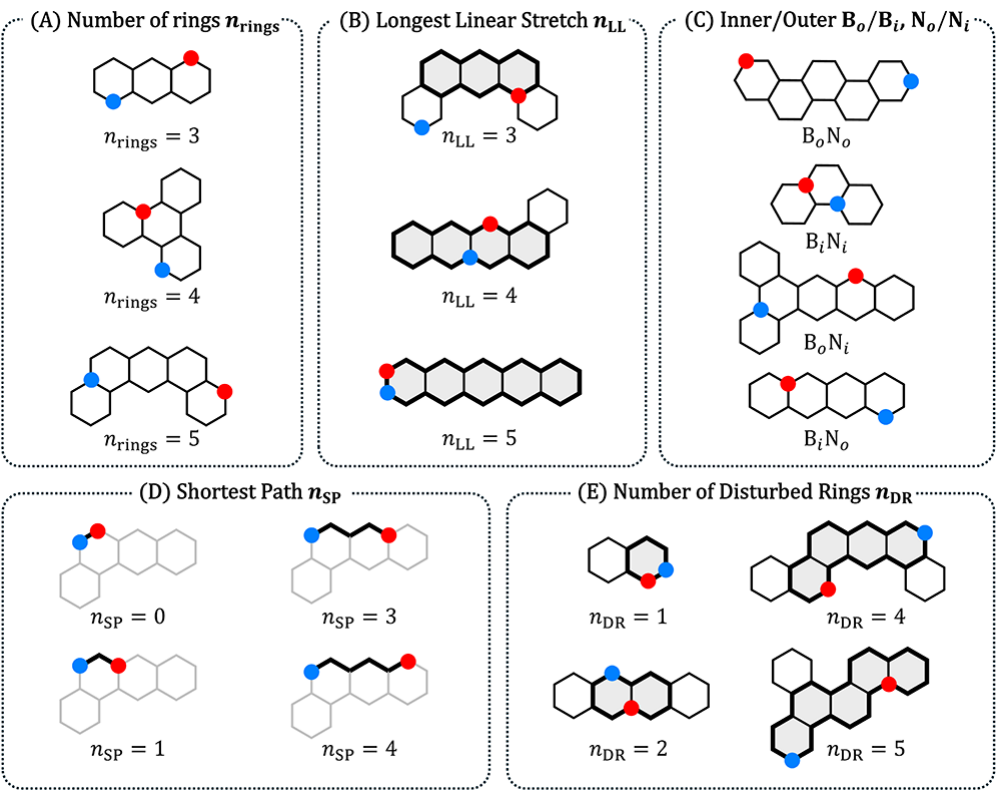
Illustration and representative examples of the five structural descriptors defined in this work. Explicit Hs and double bonds are omitted for clarity. Boron is denoted with a red circle, Nitrogen is denoted with a blue circle.

Each of these is discussed further in the next subsection.

### Structure–Property Relationships

Each of the features introduced in the previous subsection captures some aspect of the structure of a given (BN)_1_-PBH. In this subsection, we explain the rationale behind the choice of each feature and demonstrate to what extent it is relevant to the molecular properties of (BN)_1_-PBHs. For conciseness, we limit the discussion here to two properties, Δ*E*_H–L_ and *E*_rel_; the analyses performed for three additional properties (aIP, aEA, and the dipole moment) are presented and discussed in Section S5 of the Supporting Information.

#### Feature #1: *n*_rings_

Our previous investigations of PBHs and PASs^55–57^ showed that Δ*E*_H–L_ decreases as *n*_rings_ increases, which aligns with the well-known behavior of conjugated systems, whereby increasing the size of the conjugated systems raises the HOMO and lowers the LUMO. Unsurprisingly, a similar trend was observed for the (BN)_1_-PBHs (Figure 3A).

**Figure 3:**
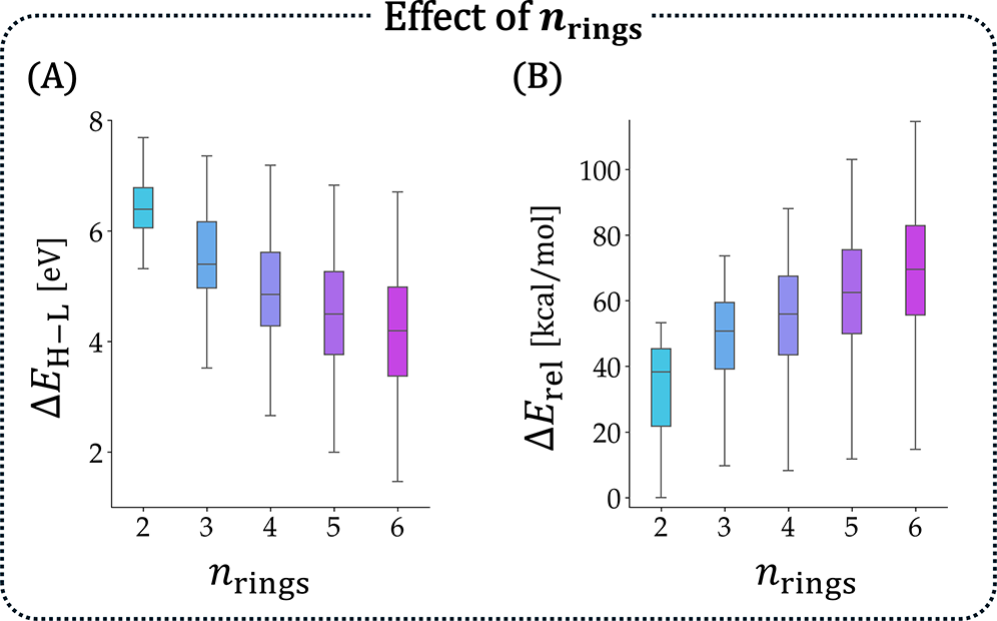
Effect of *n*_rings_ on (A) Δ*E*_H–L_ (in eV) and (B) *E*_rel_ (in kcal/mol). All molecules in COMPAS-4D were plotted.

The *E*_rel_ plots (Figure 3B) showed an opposite relationship with size: as *n*_rings_ increases, so do the maximal *E*_rel_ values. It was not surprising to observe opposite trends between Δ*E*_H–L_ and *E*_rel_; The larger Δ*E*_H–L_ values in PASs generally indicate greater aromaticity and therefore enhanced stability.^86^ In addition, there is the effect of the σ-framework. As *n*_rings_ increases, so do the various geometries that can be formed, including nonplanar substructures that incur torsional strain. As we have previously shown, there is a direct link between such nonplanar motifs (e.g., fjord, helix) and an increase in *E*_rel_ of cc-PBHs.^59,60^

We also note that, for all molecular sizes, the majority of isomers were located in the higher *E*_rel_ values (that is, less thermodynamically stable), with trailing edges toward the lower values. Visual inspection of the molecules at different areas of the distribution showed that the (BN)_1_-PBHs that appeared in the lower *E*_rel_ regions were those in which B and N shared a bond. Their greater stability could be attributed to their additional electrostatic stabilization.

#### Feature #2: *n*_LL_

In our previous analyses of cc-PBHs, we demonstrated that larger cc-PBHs can be described as sequences of angular (i.e., phenanthrene) and linear (i.e., anthracene) annulations. We showed that several molecular electronic properties are dominated by the longest consecutive sequence of linearly annelated tricycles,^59,60,75^ which we represent numerically with the *n*_LL_ feature. Each “L” represents three linearly annulated rings; thus, *n*_LL_ ≤ *n*_rings_ – 2 (*n*_LL_ = *n*_rings_ – 2 only for a fully linear isomer).

Put simply, *n*_LL_ describes the largest polyacene contained within the studied cc-PBH. To study the effect of this motif in (BN)_1_-PBHs, we plotted the distributions of the two properties, Δ*E*_H–L_ and *E*_rel_, as a function of *n*_LL_(Figure 4; to circumvent size dependency, only *n*_rings_ = 6 isomers were plotted). Figure 4A showed that Δ*E*_H–L_ did indeed decrease as *n*_LL_ increased, indicating that the underlying trend was retained despite BN-substitution. However, rather surprisingly, the relationship was substantially weaker than that observed for the parent PBHs, where *n*_LL_ was the defining feature for Δ*E*_H–L_.^55^ We further observed that *n*_LL_ had a very modest influence on *E*_rel_ (Figure 4B), which was not unexpected. Our previous investigation of the parent PBHs showed that the thermodynamic stability of these systems is mainly determined by torsional strain (i.e., nonplanar/helical motifs) and only weakly affected by the longest linear stretch. Similarly, for the (BN)_1_-PBHs all subsets had similar ranges of values, although close visual inspection of the distributions revealed that molecules with higher *n*_LL_ values were slightly less stable. Overall, these findings suggested that the longest linear stretch is weakly indicative of both the Δ*E*_H–L_ and the thermodynamic stability of (BN)_1_-PBHs. In contrast, *n*_LL_ had a very modest influence on *E*_rel_ (Figure 4B). Close visual inspection of the distributions revealed that, on average, molecules with higher *n*_LL_ values (i.e., longer linear stretches) were slightly less stable. However, all subsets had similar ranges of values. This suggested that, except for the overall extent of conjugation (assessed via *n*_rings_) the thermodynamic stability of (BN)_1_-PBHs was mainly affected by effects such as strain and electrostatics, rather than by π-effects.

**Figure 4:**
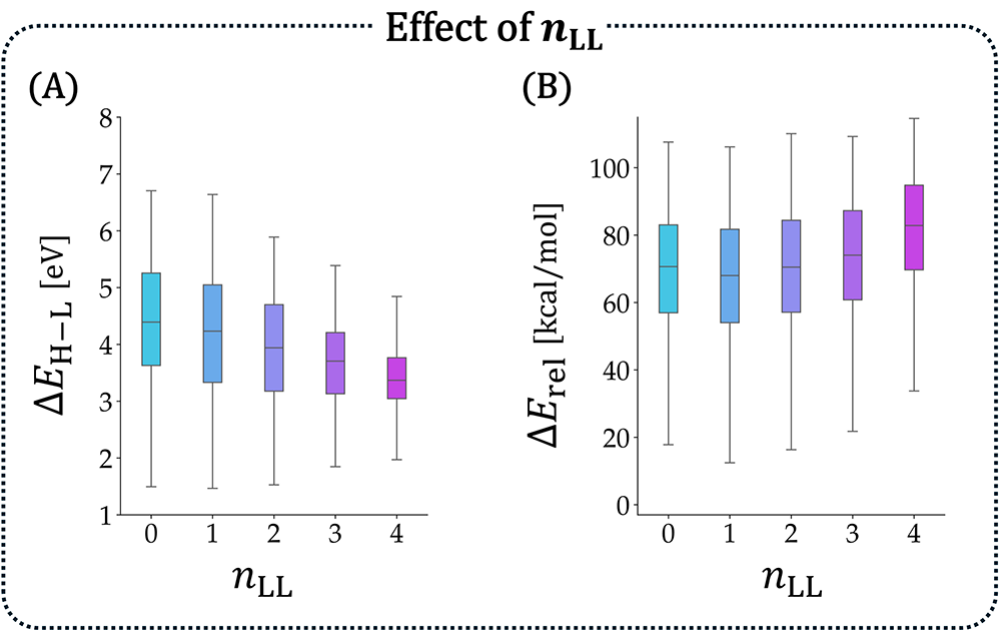
Effect of *n*_LL_ on (A) Δ*E*_H–L_ (in eV) and (B) *E*_rel_ (in kcal/mol). Only *n*_rings_ = 6 isomers are included.

#### Feature #3: B_*o*_/B_*i*_, N_*o*_/N_*i*_

To describe the location of BN-substitution within the PBH scaffold, we introduced the inner/outer classification, which indicates whether the heteroatom is located on a fused bond (i.e., inner: N_*i*_/B_*i*_) or elsewhere (i.e., outer: N_*o*_/B_*o*_). Our underlying rationale was that the molecular properties may vary when heteroatoms are on fused bonds, due to, e.g., the absence of bonded hydrogen atoms, changes in geometric strain, or their participation in two separate conjugated cycles.

To study this, we plotted the property distributions for various combinations (Figure 5; to avoid size-dependence, only *n*_rings_ = 6 isomers were included): (a) B_*o*_N_*o*_, (b) B_*i*_N_*i*_, (c) B_*o*_N_*i*_, and (d) B_*i*_N_*o*_. Somewhat disappointingly, we did not observe a strong effect, suggesting that this feature had only a mild impact on the molecular properties. For Δ*E*_H–L_, the (mild) effect was apparently important only when both heteroatoms were in the same type of position. The clearest difference was seen between the B_*o*_N_*o*_ cases (lowest values) and B_*i*_N_*i*_ cases (highest values), while essentially no differences were observed between B_*o*_N_*i*_ and B_*i*_N_*o*_.

**Figure 5:**
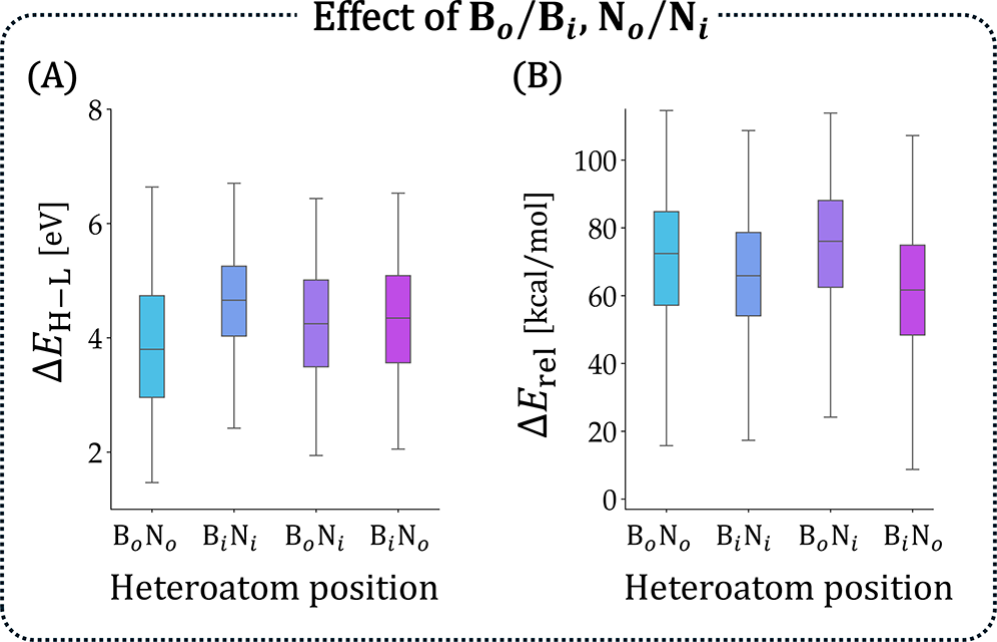
Effect of inner/outer positions on (A) Δ*E*_H–L_ (in eV) and (B) *E*_rel_ (in kcal/mol). Only *n*_rings_ = 6 isomers are included.

In contrast, for *E*_rel_, the effect appeared to be more dependent on which heteroatom was in which position. The highest *E*_rel_ values were obtained for B_*o*_N_*i*_ and the lowest for B_*i*_N_*o*_. The distribution of B_*i*_N_*i*_ was closer to B_*i*_N_*o*_, and that of B_*o*_N_*o*_ was closer to B_*o*_N_*i*_; this suggested that the position of B played a more dominant role in determining *E*_rel_. We surmised that this was due to the number and types of bonds created in each position. When B is in an inner position, it is involved in three B–C bonds; when it is in an outer position, it is involved in two B–C bonds, and another C–C bond is formed. The bond dissociation energies (BDEs) of B–C and C–C bonds are 448 and 618 kJ/mol, respectively.^87^ In contrast, B–H and C–H all have comparable BDEs (approximately 340 kJ/mol). As a result, there is a preference for C to be situated in the inner position. We note that this is just a semiquantitative analysis, based on the most rudimentary BDEs, which do not reflect the complex nature of the conjugated systems under study.

#### Feature #4: *n*_SP_

If one considers a parent PBH as a conjugated system, then substitution with a BN pair can be seen as a perturbation of the system. Even though the B and N atoms are sp^2^-hybridized and the overall conjugation is formally retained, we hypothesized that the difference in electronegativity creates a disturbance in the original polyene conjugation. Simply put, the dominant substructure in the molecule becomes the polyene-like conjugated path between the B and N atoms. Hence, the molecular properties should be directly dependent on the distance between B and N. This led us to define the *n*_SP_ feature, which is the number of carbons between the B and N atoms, along the shortest possible acyclic path (several examples are shown in Figure 6).

**Figure 6:**
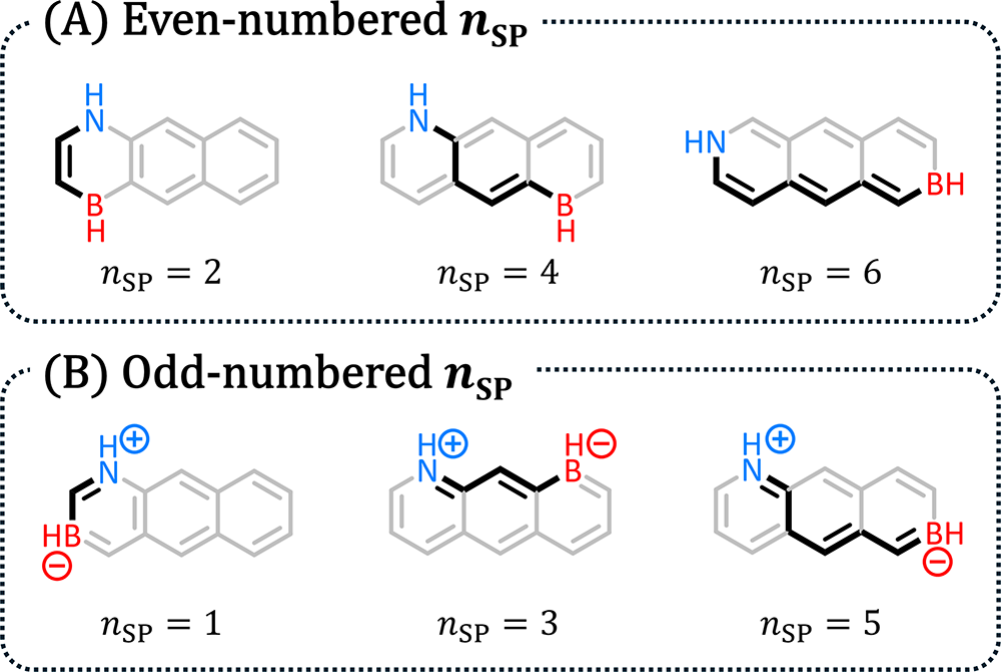
Representative examples of molecules with (A) even-numbered and (B) odd-numbered *n*_SP_ values.

The distributions of Δ*E*_H–L_ and *E*_rel_ against *n*_SP_ showed that this feature did indeed have a strong effect on the molecular properties (Figure 7; to avoid size-dependence, only *n*_rings_ = 6 isomers were included). The Δ*E*_H–L_ decreased and the *E*_rel_ increased with greater *n*_SP_ values. Interestingly, the Δ*E*_H–L_ plot was reminiscent of the well-known 1/*n* behavior of polyenes, which supported the idea that the path between B and N is indeed the operative polyene structure, as we hypothesized. In both plots, we noticed a minor “zigzag” effect between odd- and even-numbered *n*_SP_ values, which suggested that they were actually two separate series, which could be explained with resonance structures (RSs). Molecules with even *n*_SP_ (Figure 6A) had homologous vinylic configurations between the B and N, while molecules with odd *n*_SP_ (Figure 6B) had homologous allylic configurations. Similar trends were observed for aIP and aEA (see Section S5 in the Supporting Information).

**Figure 7:**
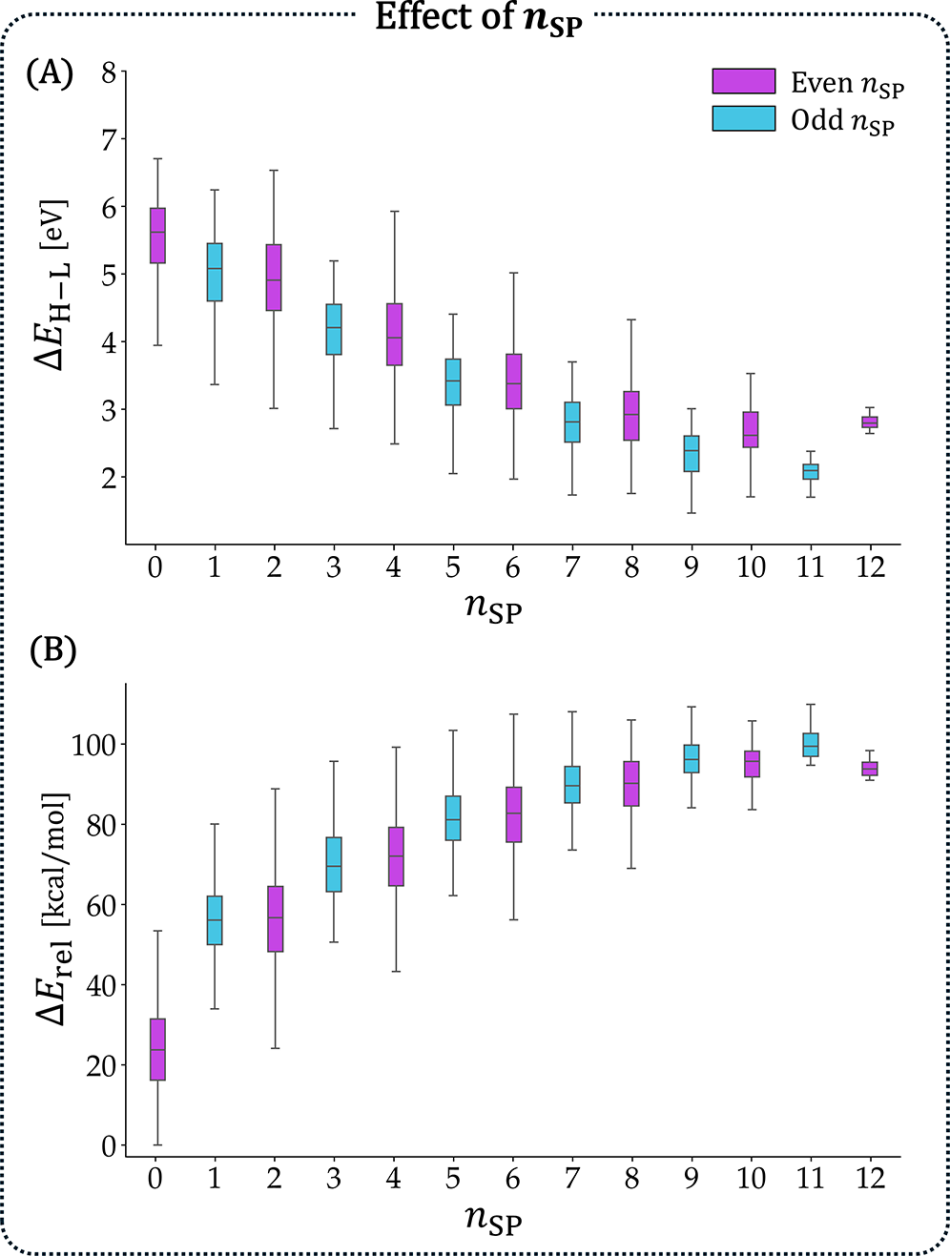
Effect of *n*_SP_ on (A) Δ*E*_H–L_ (in eV) and (B) *E*_rel_ (in kcal/mol). Only *n*_rings_ = 6 isomers are included.

We also noted that the *E*_rel_ values for *n*_SP_ = 0 were substantially lower than the rest of the series, which could be explained by the strong electrostatic stabilization that occurred when B and N shared a bond. This important characteristic was not captured directly by any of the other features. Indeed, this substantial effect has led some researchers to refer to the B–N bond as “the smallest p–n junction”.^88^

#### Feature #5: *n*_DR_

In addition to the perturbation of the polyene conjugation, there was also another type of perturbation to consider: the disruption of cyclic conjugation (i.e., aromaticity) in the polycyclic scaffold. Aromaticity in PBHs is often evaluated by the number of Clar sextets (i.e., disjoint sets of 6 π-electrons) in the molecule—the greater the number of sextets, the “more aromatic” the molecule.^89^

As shown in Figure 8A, it is possible to draw two types of RSs for a ring containing B/N: quinoidal or Clar. In the former, there is no Clar sextet (i.e., no aromatic character) and no formal charges on either heteroatom. In the latter, a Clar sextet indicates the existence of aromatic character but comes at the cost of forming formal charges on the B and N atoms. Despite the existence of a Clar sextet, the aromaticity of such rings is attenuated due to the strong localization of π-electrons, as is known from the example of borazine.^90–92^ Thus, incorporation of B/N into a ring necessarily disrupts its aromaticity.

**Figure 8:**
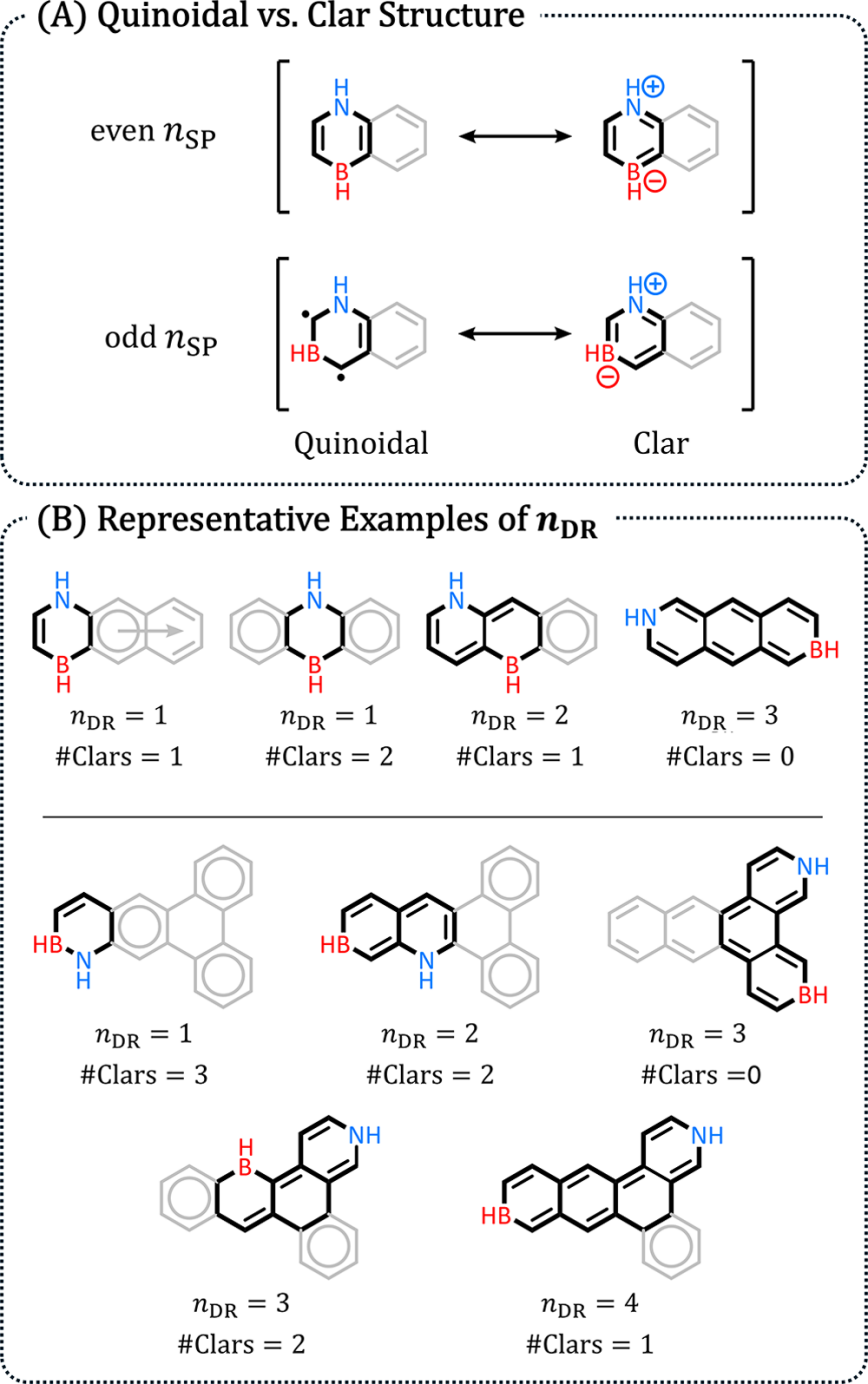
(A) Quinoidal and Clar resonance structures for (BN)_1_-PBHs with an even (top) and odd (bottom) *n*_SP_ value. (B) Two examples of different *n*_DR_ values for different (BN)_1_-PBH isomers stemming from the same scaffold.

The disruption is exacerbated when B and N are located in different rings, because any rings situated between them will also adopt a quinoidal structure, precluding them from forming Clar sextets (see examples in Figure 8B). Therefore, the positions of B and N within the scaffold are expected to have a direct effect on the number and size of conjugated circuits, which in turn have a direct impact on the stability (*E*_rel_) and Δ*E*_H–L_ of the (BN)_1_-PBH. To represent this as a numerical feature, we defined *n*_DR_, which is the number of disrupted rings. We note that *n*_DR_ is not the sole parameter affecting the number of Clar sextets formed; the geometry of the scaffold also plays an important role. Nevertheless, there is a qualitative correspondence, which led us to include *n*_DR_ in our feature set.

This choice was validated by the plots of Δ*E*_H–L_ and *E*_rel_ versus *n*_DR_, which revealed strong relationships between the two properties and this structural feature. Figure 9A showed that Δ*E*_H–L_ steadily decreased with *n*_DR_. This result aligned with our rationalization because the “more aromatic” (i.e., less disrupted) a system was, the greater its Δ*E*_H–L_ was expected to be. Indeed, it has been experimentally determined that the absorption wavelength decreases for isomers with higher numbers of Clar sextets.^93^ For the same reason, the decrease in *E*_rel_ with greater *n*_DR_ values was unsurprising (Figure 9B). Aromaticity is a stabilizing property, hence, the more it is disrupted, the less thermodynamically stable the molecule should be.

**Figure 9:**
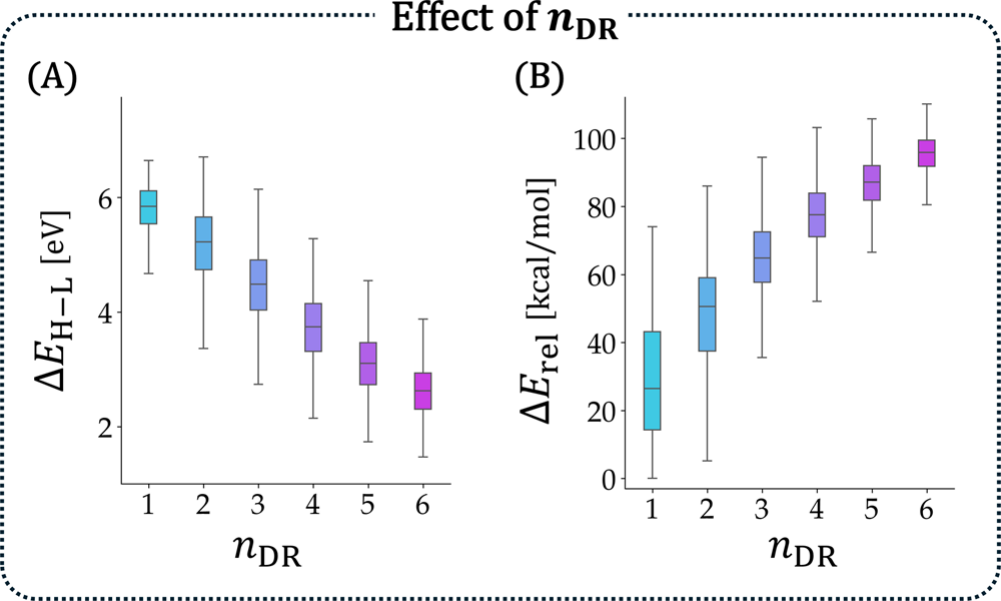
Effect of *n*_DR_ on (A) Δ*E*_H–L_ (in eV) and (B) *E*_rel_ (in kcal/mol). Only *n*_rings_ = 6 isomers are included.

## Predictive Performance of Feature Set

Having defined our set of simple yet meaningful structural features, we turned to the next phase: using these features as input for predictive models. Though each of the features showed a certain relationship to the properties of the (BN)_1_-PBHs, none of them was sufficient on its own to provide a quantitative prediction of those same properties. However, the combination of these features could enable an accurate prediction of molecular properties. If so, this would validate our selection of structural motifs. Moreover, a successfully trained model could be interrogated to reveal the relative importance of the different features.

To this end, we trained several regression models to predict either the Δ*E*_H–L_ or the *E*_rel_. In all cases, the molecular structures were inputted as a vector comprising the five features detailed above. No other structural or property data were used to train the models. A 75:25 (train/test) data split and 5-fold cross-validation were used. The results of the best-performing model, the Light Gradient-Boosting Method (LGBM), are presented in Figure 10.

**Figure 10:**
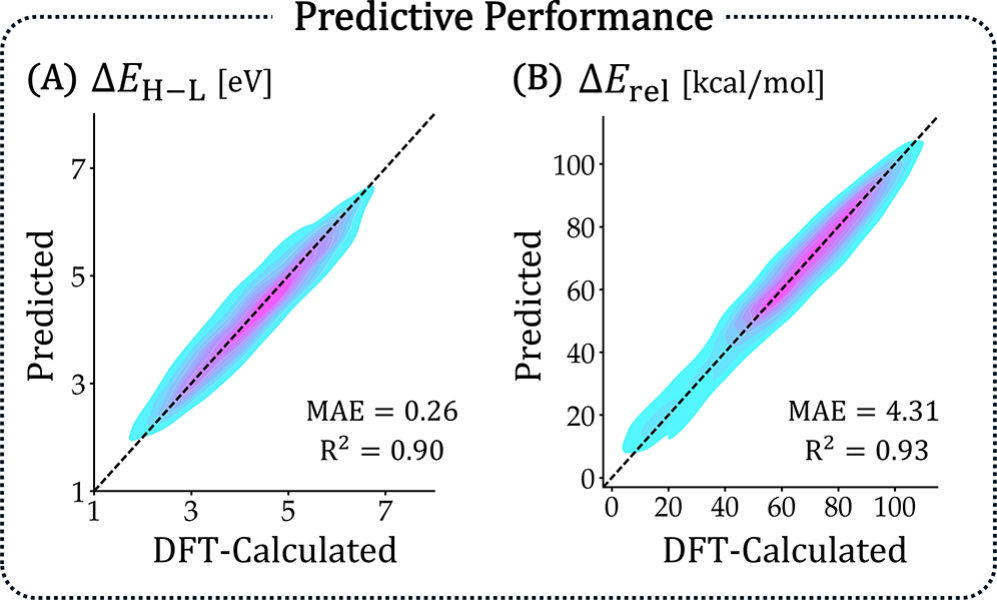
Performance of the trained LGBM regressor models for prediction of (A) Δ*E*_H–L_ and (B) *E*_rel_. Each plot contains the predicted vs calculated values of the 25% of data reserved for testing. MAE and *R* ^2^ are noted on each plot. MAE for Δ*E*_H–L_ is in eV and for *E*_rel_ in kcal/mol.

Considering the simplicity of our feature set (including the fact that none of the features required any preliminary calculations), the performance of the LGBM model was remarkable. The mean absolute error (MAE) for Δ*E*_H–L_ was MAE = 0.26 eV (with *R* ^2^ = 0.90) and for *E*_rel_ was MAE = 4.31 kcal/mol (*R* ^2^ = 0.93) (Figure 10). The other models performed comparably well, providing further evidence of this feature set’s utility. Those results, as well as an ablation study demonstrating the importance of each feature, are provided in Section S6 of the Supporting Information.

### Conclusions

The chemical space of BN-PBHs is vast and complex. To date, only a small number of molecules have been synthesized, yet these have already shown promise for (opto)­electronics. A methodical and comprehensive exploration is needed to identify additional promising candidates but, more importantly, to define principles for the molecular design of new functional BN-PBHs.

In this study, we reported on the data-driven exploration of the chemical compound space of (BN)_1_-PBHs containing up to six rings—a collection of 24k molecules. To this end, we computationally generated two new data sets, COMPAS-4D and COMPAS-4x, which contain the geometries and properties of these molecules obtained at the CAM-B3LYP/def2-SVP and GFN1-xTB levels of theory, respectively.

To establish principles for the design of new functional (BN)_1_-PBHs with specific properties, we sought to identify dominant structure–property relationships. Based on our domain expertise in PASs, we defined a small set of structural motifs: Two features described the size and geometry of the scaffold (*n*_rings_ and *n*_LL_) and the other three addressed the location of B and N within the scaffold (B_*o*_/B_*i*_, N_*o*_/N_*i*_, *n*_SP_, and *n*_DR_). An important aspect of our defined features was that none of them required any quantum chemical calculations, including geometry optimization. In other words, they were truly “back-of-the-envelope”.

We investigated the effect of each feature on a set of molecular properties (Δ*E*_H–L_, *E*_rel_, aIP, aEA, and dipole moment) and found clear trends that shed light on the underlying relationships. Importantly, the observed trends could be rationalized using fundamental chemical concepts, such as RSs and aromaticity. Notably, the strongest effect was observed for *n*_DR_. This result highlighted the importance of considering PASs through the lens of intuitive, organic chemistry-based concepts.

Finally, the defined features were used as input to train various models. The models showed remarkable predictive performance, especially considering the small number of features and their simplicity. This success demonstrated the power of chemically informed feature engineering. Most importantly, the intuitive, simple, and interpretable nature of these features allows one to rationally design new (BN)_1_-PBHs based on an understanding of the underlying structure–property relationships.

## Supplementary Material



## Data Availability

The data generated in the course of this study and the jupyter notebooks used for analysis and plot generation are all available at https://gitlab.com/porannegroup/compas/-/tree/main/COMPAS-4?ref_type=headshttps://gitlab.com/porannegroup/compas. A minted version of the data reported in this manuscript is available on Zenodo (10.5281/zenodo.15356092https://doi.org/10.5281/zenodo.15356092) (data set posted on 2025–05–07).

## Notes

Computational details, input templates, overview of the COMPAS-4D and COMPAS-4x data sets, feature correlation analysis, analysis of three more properties (aIP, aEA, dipole moment), regression analyses for additional models (PDF)
