# Supplementary material for: COMPAS-4: A Data Set of (BN)1 Substituted Cata-Condensed Polybenzenoid Hydrocarbons—Data Analysis and Feature Engineering
Source: J Chem Inf Model. 2025 May 23;65(11):5508–17. doi: 10.1021/acs.jcim.5c00608 (PMC12152957; doi:10.1021/acs.jcim.5c00608)
Supplement: Supplementary file 1 [file ci5c00608_si_001.pdf]

Supporting Information

COMPAS-4: a Dataset of $(BN)_1$ Substituted *Cata*-Condensed Polybenzenoid Hydrocarbons – Data Analysis and Feature Engineering

Sabyasachi Chakraborty,[a] Itay Almog,[a] and Renana Gershoni-Poranne[a]

[a] *The Schulich Faculty of Chemistry and the Resnick Sustainability Center for Catalysis, Technion – Israel Institute of Technology, Haifa 32000, Israel*

e-mail:rporanne@technion.ac.il

Contents

S1 Computational Methods and Input Templates **S4**

S1.1 xTB calculations for COMPAS-4x S4

S1.2 DFT calculations for COMPAS-4D S4

S2 Benchmarking **S5**

S2.1 Semi-empirical S5

S2.2 DFT S6

S3 Feature Correlation **S8**

S4 Overview of COMPAS-4x and COMPAS-4D **S9**

S4.1 Structural distributions for COMPAS-4x and COMPAS 4D S9

S4.2 Property distributions for COMPAS-4x and COMPAS 4D S10

S5 Effect of selected features on additional properties **S12**

S5.1 Feature #1: Number of rings (n_{rings}) S12

S5.2 Feature #2: Longest Linear Stretch (n_{LL}) S13

S5.3 Feature #3: B_i/B_o, N_i/N_o S14

S5.4 Feature #4: Shortest Path (n_{SP}) S15

S5.5 Feature #5: Number of disturbed rings n_{DR} S17

S6 Regression analyses **S18**

S6.1 Regression protocol S18

S6.2 Performance of different models on $\Delta E_{\text{H-L}}$ and E_{rel} S19

S6.3 aIP, aEA, and μ S20

S6.4 Ablation studies S21

S7 References **S23**

List of Tables

S1 Comparison of experimentally measured absorption wavelengths and DFT-calculated $\Delta E_{\text{H-L}}$ values(CAM-B3LYP/def2-SVP) for a selection of $(\text{BN})_1$-PBHs. Superscripts next to experimental values indicate references from which these values were collected. All values are in eV. S6

List of Figures

S1 Scatter plots of HOMO and LUMO computed at CAM-B3LYP/def2-SVP from COMPAS-2 and at semi-empirical levels. S5

S2 Scatter plot of $\Delta E_{\text{H-L}}$ versus experimentally measured absorption wavelengths for a selection of $(\text{BN})_1$-PBHs. S7

S3 Correlation plot of all features tested. The value in each cell is the R^2 computed for each feature when tested on the COMPAS-4D dataset. S8

S4 Structural distributions in COMPAS-4D. S9

S5 Structural distributions in COMPAS-4x. S9

S6 Property distributions of $(\text{BN})_1$-PBHs in COMPAS-4x and COMPAS-4D. . S10

S7 Scatter plots of COMPAS-4x versus COMPAS-4D properties. S10
S8 Scatter plots of COMPAS-1x versus COMPAS-1D properties ($2 \leq n_{\text{rings}} \leq 10$, 8,677 data points). S11
S9 Scatter plots of COMPAS-1x versus COMPAS-1D properties ($2 \leq n_{\text{rings}} \leq 6$, 57 data points). S11
S10 The effect of n_{rings}on A) aIP (in kcal/mol), B) aEA(in kcal/mol), and C) dipole moment μ (in Debye). For aIP and aEA, molecules with negative frequencies in their charged states were ignored. S12
S11 The effect of n_{LL} on A) aIP (in kcal/mol), B) aEA (in kcal/mol), and C) dipole moment μ (in Debye). For aIP and aEA, molecules with negative frequencies in their charged states were ignored. Also, only n_{rings}=6 isomers are included. S13
S12 The effect of inner/outer positions on A) aIP (in kcal/mol), B) aEA (in kcal/mol), and C) μ (in Debye). Only n_{rings}=6 isomers are included. S14
S13 The effect of n_{SP}on A) aIP (in kcal/mol), B) aEA (in kcal/mol), and C) dipole moment μ (in Debye). For aIP and aEA, molecules with negative frequencies in their charged states were ignored. Also, only n_{rings}=6 isomers are included. S15
S14 Schematic showing how dipole vector deviation (θ) from heteroatom orientation was calculated. S16
S15 Stacked histogram of deviations of the dipole vector from the B to N orientation vector in COMPAS-4D classified based on n_{SP}. S16
S16 The effect of n_{DR} on A) aIP (in kcal/mol), B) aEA (in kcal/mol), and C) dipole moment μ (in Debye). Only n_{rings}=6 isomers are included. S17
S17 $\Delta E_{\text{H-L}}$ as predicted by Random Forest Regression (RF), CatBoost Regression (CatBoost), and linear regression (LR). Also provided are the Mean absolute error (MAE), and coefficient of determination (R^2) for the final models. All values are in eV. S19
S18 E_{rel} as predicted by Random Forest Regression (RF), CatBoost Regression (CatBoost), and linear regression (LR). Also provided are the Mean absolute error (MAE), and coefficient of determination (R^2) for the final models. All values are in kcal/mol. S19
S19 Performance of LGBM regressor models for prediction of A) aIP, B) aEA, and C) μ. S20
S20 Performance of RF regressor models for prediction of A) aIP, B) aEA, and C) μ. S20
S21 Performance of CatBoost regressor models for prediction of A) aIP, B) aEA, and C) μ. S21
S22 Performance of LR models for prediction of A) aIP, B) aEA, and C) μ on the test set (25% of data). MAE and R^2 are noted on each plot. MAEs for aIP and aEA are in kcal/mol; MAE for μ is in Debye. S21
S23 LGBM ablation study for $\Delta E_{\text{H-L}}$. S22
S24 LGBM ablation study for E_{rel}. S22

S1 Computational Methods and Input Templates

In this section we provide the templates and command line instructions used to generate the electronic properties of COMPAS-4D and COMPAS-4x. All semiempirical calculations were performed at the GFN1-xTB level with xTB version 6.2. All DFT calculations were performed with ORCA 5.0.3 and 5.0.4.

S1.1 xTB calculations for COMPAS-4x

xTB calculations were launched from the command line using the following command template:

```
Path\to\xTB\executable\xtb file.xyz --ohess --chrg m --namespace file -gfn
↪ 1 > output
```

Here, `m` stands for the requested charge and `file.xyz` contains the Cartesian Coordinates of the molecule with the number of atoms and the title occupying the first and second lines, respectively. `namespace` and `output` are user-defined variables.

S1.2 DFT calculations for COMPAS-4D

For geometry optimization, the following input template was used, where `Cartesian Coordinates` was replaced by the actual coordinates of the molecule. For neutral molecule calculations–`m=0` and `n=1`, for cation calculations–`m=1` and `n=2`, and for anion calculations–`m=-1` and `n=2`.

```
!CAM-B3LYP D3BJ def2-SVP def2/J RIJCOSX TightSCF TightOpt Freq
*xyz m n
Cartesian Coordinates
Element x y z
*
%MaxCore 6400
%scf
 Convergence VeryTight
end
%pal nprocs 8
end
%geom
 MaxIter 250
end
```

S2 Benchmarking

The computational methods were selected following benchmarking procedures, which were reported previously.[1,2] In this section, we reiterate those findings and provide some additional validation of the chosen methods.

S2.1 Semi-empirical

In our previous investigations of cc-PBHs, we found that GFN2-xTB shows excellent agreement with DFT. However, when we investigated heterocyclic systems,[2], we found that the same method did very poorly in evaluating the HOMO energies (see Figure S1). We compared the results of GFN1-xTB, GFN2-xTB, PM6, and PM7 in order to select a method that is sppropriate for heterocyclic PASs. Based on this analysis, GFN1-xTB was chosen, as it shows the best agreement with DFT results.

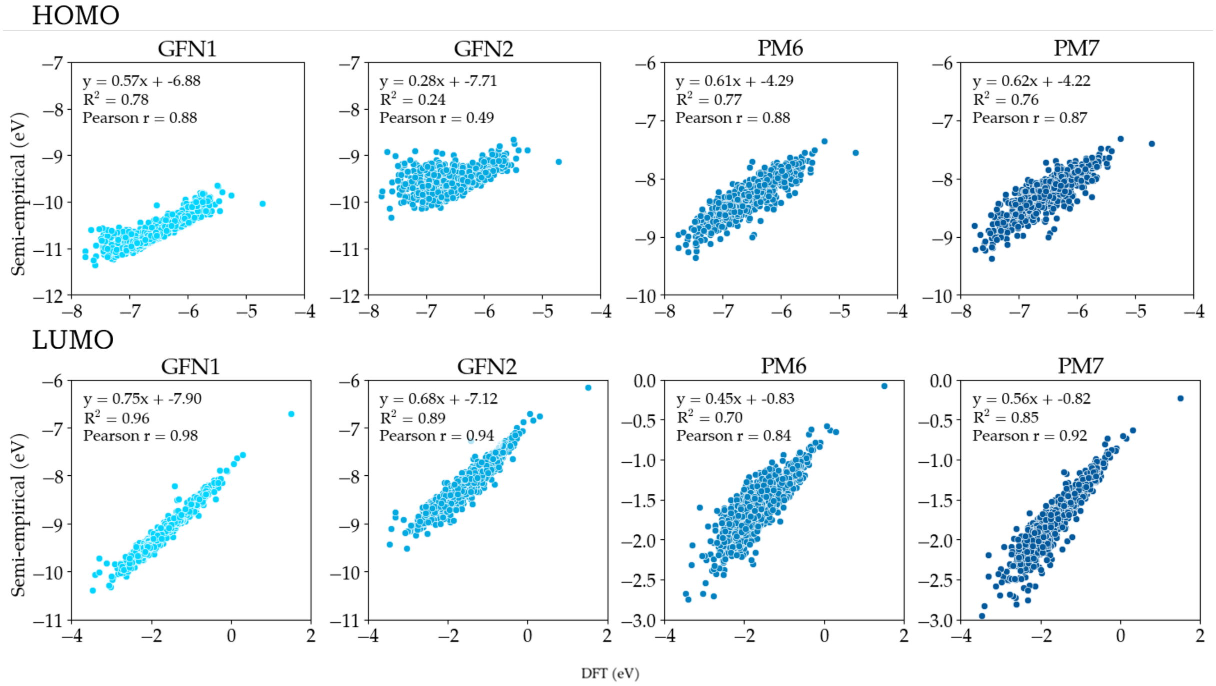

Figure S1: Scatter plots of HOMO and LUMO computed at CAM-B3LYP/def2-SVP from COMPAS-2 and at semi-empirical levels.

S2.2 DFT

The CAM-B3LYP functional and def2-SVP basis-set were chosen because they provided a satisfactory balance between computational resources and accuracy.

Accuracy. We found excellent agreement between experimental values of absorption wavelengths (which indirectly relate to $\Delta E_{\mathrm{H-L}}$ values), $S_0 - S_1$ and $S_0 - T_1$ energy gaps and computed values of these properties for cc-PBHs.[3,4] For COMPAS-4, we extracted additional absorption wavelengths for a selection of $(\mathrm{BN})_1$-PBHs (Table S1) and compared them with our calculated values. The correlation is shown in Figure S2.

Table S1: Comparison of experimentally measured absorption wavelengths and DFT-calculated $\Delta E_{\mathrm{H-L}}$ values(CAM-B3LYP/def2-SVP) for a selection of $(\mathrm{BN})_1$-PBHs. Superscripts next to experimental values indicate references from which these values were collected. All values are in eV.

$(\mathrm{BN})_1$-PBHs	Expt.	CAM-B3LYP/ def2-SVP
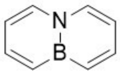	4.13[5]	7.69
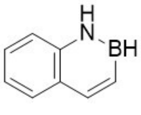	3.85[5]	7.60
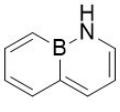	3.21[5]	6.60
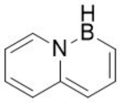	3.10[5]	6.59
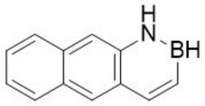	3.22[6]	6.38
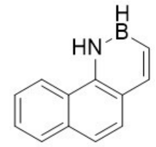	3.53[7]	6.91
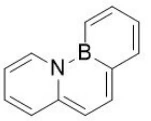	2.78[8]	5.85
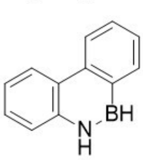	3.80[8]	7.35
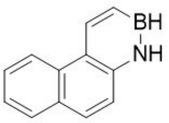	3.59[9]	6.82

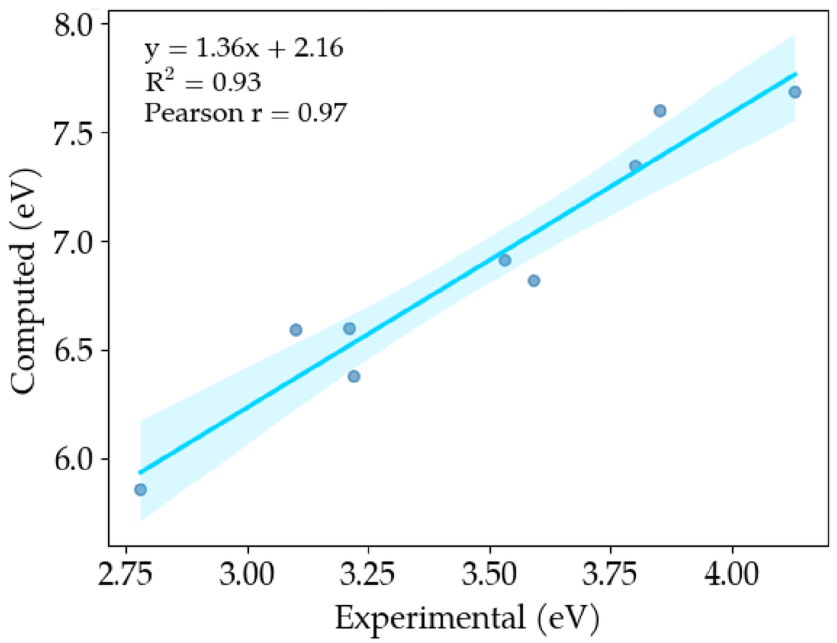

Figure S2: Scatter plot of $\Delta E_{\mathrm{H-L}}$ versus experimentally measured absorption wavelengths for a selection of $(BN)_1$-PBHs.

Computational cost. As we previously reported[1] B3LYP proved more robust than PBE0, which did not converge in ~13% of the optimization calculations. The def2-SVP basis-set showed excellent agreement with def2-TZVP, at a fraction of the computational time.

S3 Feature Correlation

Our study identified five intuitive and simple structural features of $(BN)_1$-PBHs:

- Feature #1: n_{rings}
- Feature #2: n_{LL}
- Feature #3: B_i/B_o, N_i/N_o
- Feature #4: n_{SP}
- Feature #5: n_{DR}

In Figure S3, we present a matrix that evaluates the correlation between the different features. The goal is to have features that have low to no correlation with each other. This ensures that each feature captures a different aspect of the chemical information contained in the molecular structure. Conversely, if features are highly correlated, the same information is being provided, which is redundant. This can also have an impact on the models trained on the features. For linear models (e.g., linear regression or logistic regression), multicollinearity can yield possibly numerically unstable solutions. More importantly, it skews the interpretation of the importance of the different features.

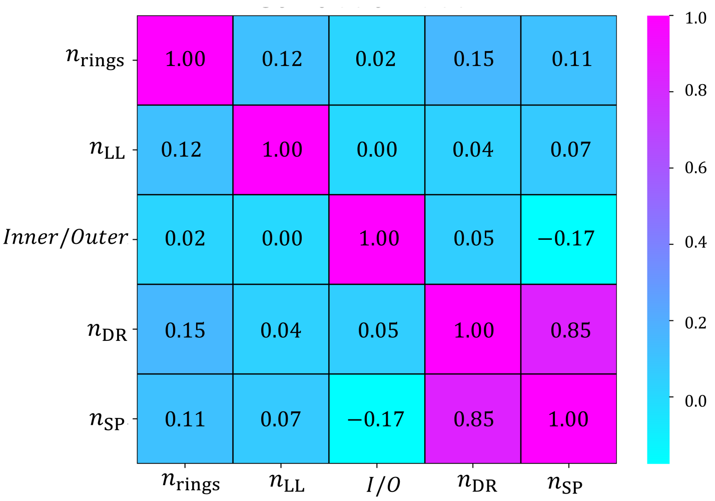

Figure S3: Correlation plot of all features tested. The value in each cell is the R^2 computed for each feature when tested on the COMPAS-4D dataset.

From Figure S3, it is clear that all our features present a low degree of correlation with each other. The only two features that show a high degree of correlation are n_{SP} and n_{DR}, which may be expected given that they are both dependent on the relative distance between B and N. In subsequent sections, we show via an ablation study that both of these features are important and necessary for successful property prediction for $(BN)_1$-PBHs.

S4 Overview of COMPAS-4x and COMPAS-4D

S4.1 Structural distributions for COMPAS-4x and COMPAS 4D

Figures S4 and S5 display the geometric distributions of the optimized geometries in COMPAS-4D and COMPAS-4x, respectively, demonstrating that the two datasets are highly similar. As shown in the top rows, the majority of species are along the I_2/I_3 axis, which indicates linear or planar structures. Only a small fraction of the molecules show "three-dimensional" geometry, which generally comes from helical motifs, as corroborated by the histograms (bottom rows). The geometry is mostly determined by the PBH scaffold, with slight variations that stem from the inclusion of B and N atoms. The addition of charge does not alter this structural distribution.

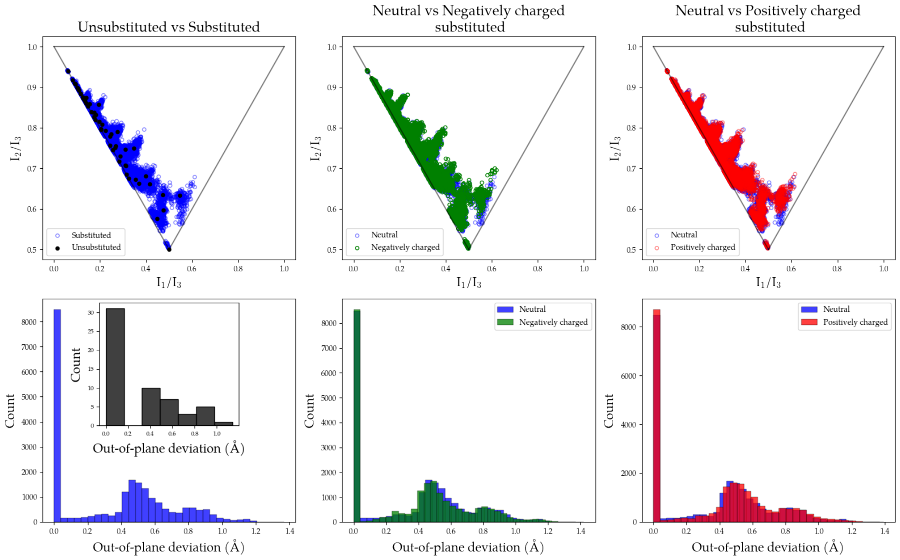

Figure S4: Structural distributions in COMPAS-4D.

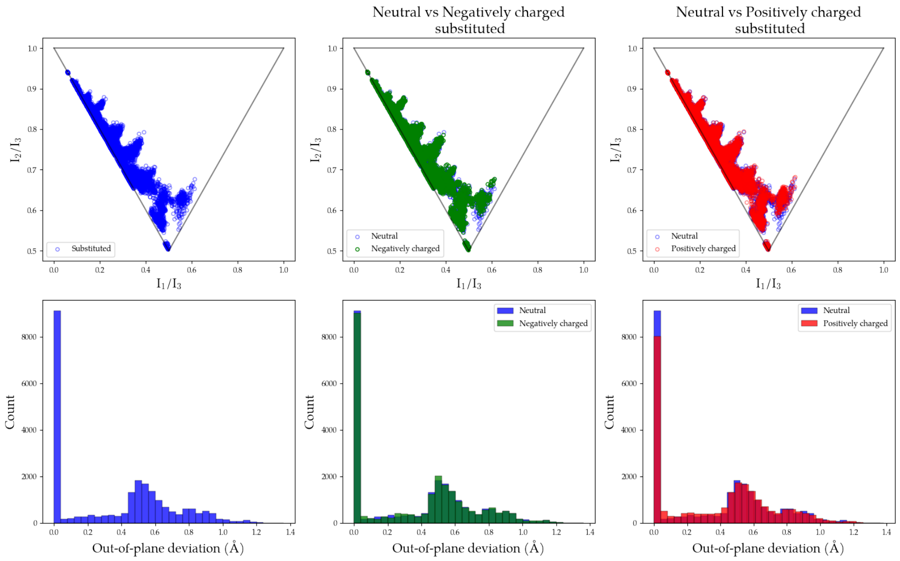

Figure S5: Structural distributions in COMPAS-4x.

S4.2 Property distributions for COMPAS-4x and COMPAS 4D

Figure S6 shows the distributions of properties in COMPAS-4x and COMPAS-4D. Nevertheless, the scatter plots in Figure S7 show that there is a good agreement between them. Although visual inspection suggests that the correlations are not necessarily linear for all properties (e.g., the lower range of $\Delta E_{\mathrm{H-L}}$ values has a distinct broadening and apparent change of slope), statistical metrics (Pearson's correlation coefficient, ρ, and the coefficient of determination, R^2) both indicate that the data do follow linear fittings. (Note: All molecules with imaginary frequencies in their charged states were omitted (total of 22,282 data points)).

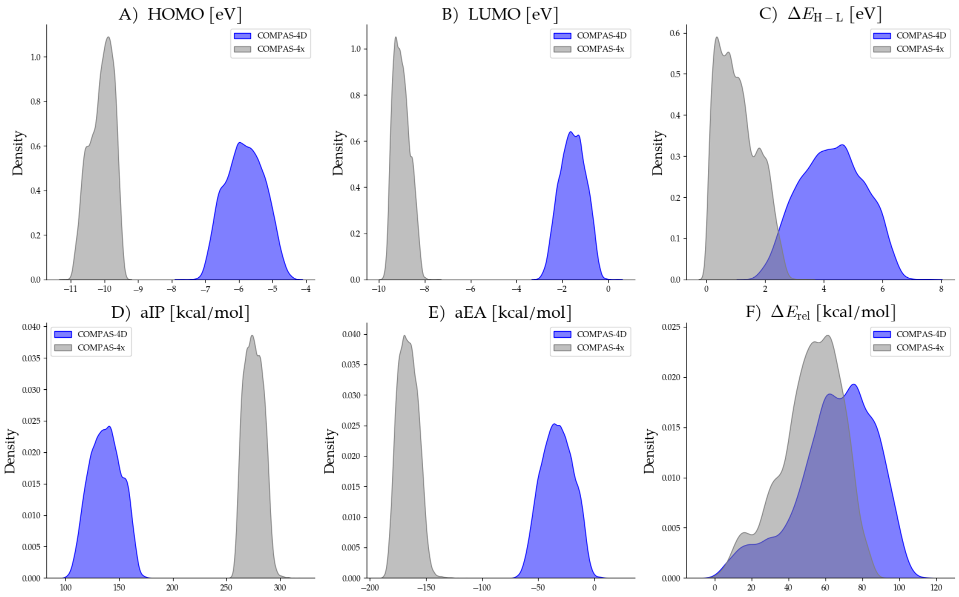

Figure S6: Property distributions of $(BN)_1$-PBHs in COMPAS-4x and COMPAS-4D.

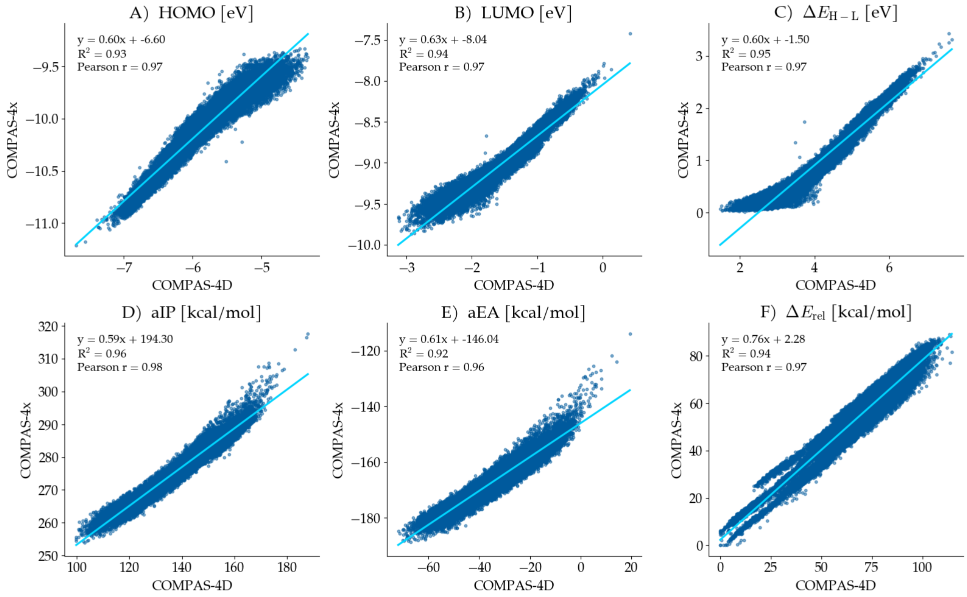

Figure S7: Scatter plots of COMPAS-4x versus COMPAS-4D properties.

For comparison, we include identical analyses performed on COMPAS-1 data. Figure S8) shows the scatter plots for all molecules in the COMPAS-1D dataset ($2 \leq n_{\text{rings}} \leq 10$). Figure S9 shows the scatter plots for the $2 \leq n_{\text{rings}} \leq 6$ molecules in COMPAS-1D.

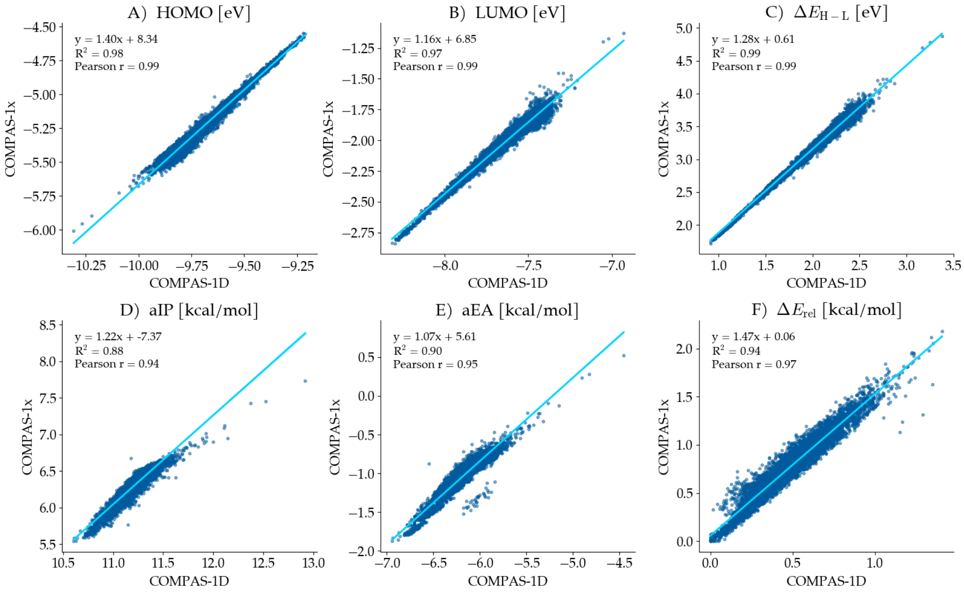

Figure S8: Scatter plots of COMPAS-1x versus COMPAS-1D properties ($2 \leq n_{\text{rings}} \leq 10$, 8,677 data points).

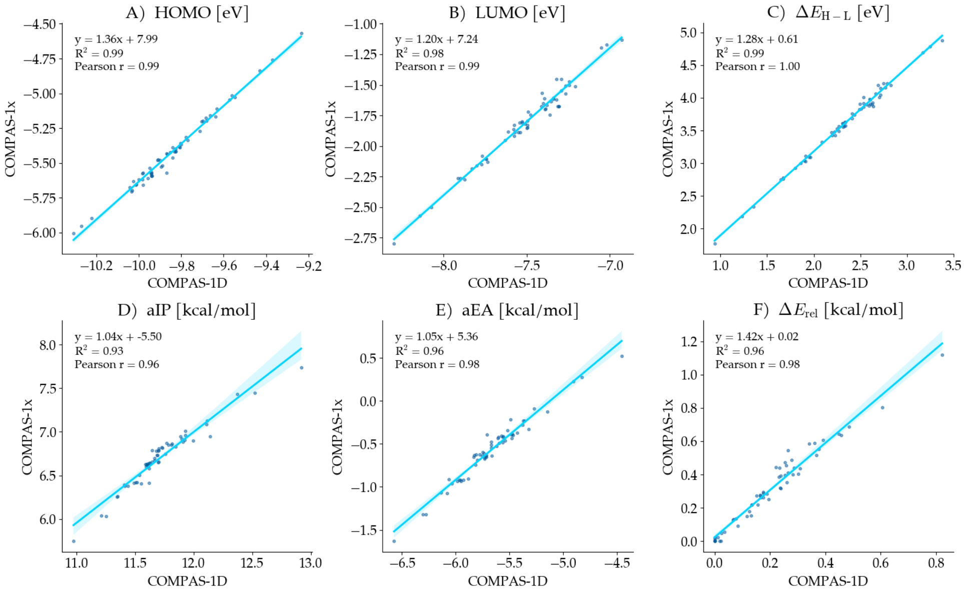

Figure S9: Scatter plots of COMPAS-1x versus COMPAS-1D properties ($2 \leq n_{\text{rings}} \leq 6$, 57 data points).

S5 Effect of selected features on additional properties

In the main text, we describe the structure-property relationships between our set of five features. This section presents the structure-property relationships between the same set of features and three additional molecular properties: aIP, aEA, and the dipole moment (μ). The subsections are arranged in the same order as they are discussed in the main text. All properties discussed are from COMPAS-4D.

S5.1 Feature #1: Number of rings (n_{rings})

$\Delta E_{\text{H-L}}$ and E_{rel} are indirectly dependent on the size of the molecule (*via* the extent of conjugation and aromaticity). In contrast, aIP, aEA, and μ have a more direct relationship. The first two are both size-dependent properties because the stability of the charged species is related to the ability of the molecule to delocalize charge—hence, larger systems will enjoy better stability. This is apparent in Figure S10, where with increasing size, the $(BN)_1$-PBH stabilizes charges well, resulting in diminishing aIP and aEA.

μ is also size-dependent, as it depends on the distance between the $\delta+$ and $\delta-$ in the molecule, which for $(BN)_1$-PBHs corresponds to the positions of the N and B atoms, respectively. The larger the molecule is, the larger this distance can potentially be. However, even though higher n_{rings} values provide the *opportunity* for larger μ, this does not mean that all large molecules have larger μ values. If B and N are close to one another, μ can be small, regardless of molecule size. This is exactly was we observe in Figure S10: as n_{rings} increases, the distribution broadens the maximal μ shifts upward. The shifting of the distribution is due to the minority of $(BN)_1$-PBHs exhibiting high μ values.

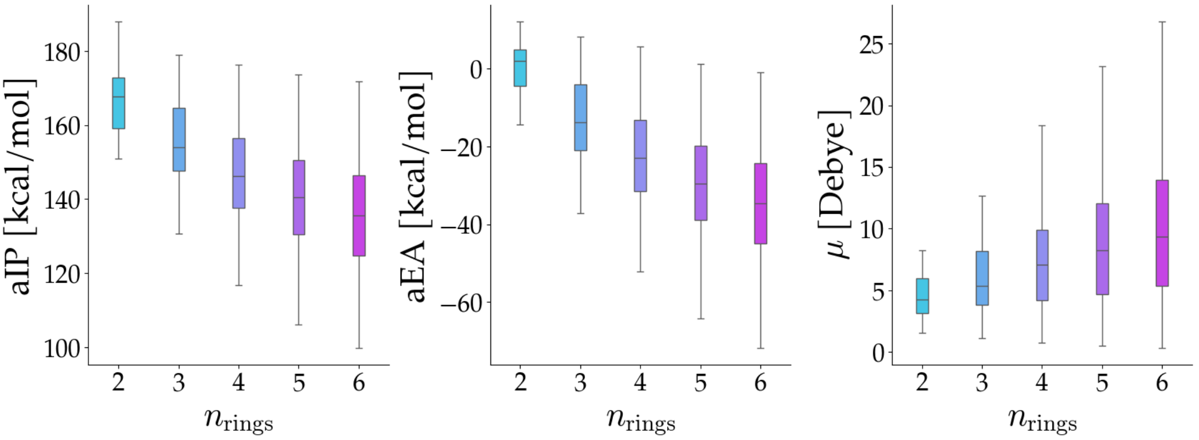

Figure S10: The effect of n_{rings}on A) aIP (in kcal/mol), B) aEA(in kcal/mol), and C) dipole moment μ (in Debye). For aIP and aEA, molecules with negative frequencies in their charged states were ignored.

Overall, the number of rings in a $(BN)_1$-PBH is a chemically intuitive and easily accessible feature, and it captures the underlying trends of many molecular electronic properties. Having just established that n_{rings} plays a non-negligible role, the following four subsections that investigate the influence of the other structural features will exclusively focus on the n_{rings}=6 subspace.

S5.2 Feature #2: Longest Linear Stretch (n_{LL})

Similar to n_{rings}, a decrease in aIP and aEA is also observed with increasing values of n_{LL}. According to Koopmans' theorem, aIP is directly related to the energy of the HOMO, and aEA is directly related to the energy of the LUMO. Since both of the MO energies are tied to n_{LL}(as we have previously shown), it is not surprising to find the relationship extended to aIP and aEA, as seen in Figure S11. For μ, we observe that the distribution hardly changes as n_{LL} increases. This is not surprising, considering all of the molecules included in this analysis are of the same size (n_{rings}=6), and the distance between B and N does not rely on the presence of linear stretches. Therefore, the average stays constant, and each subset includes trailing edges towards higher values.

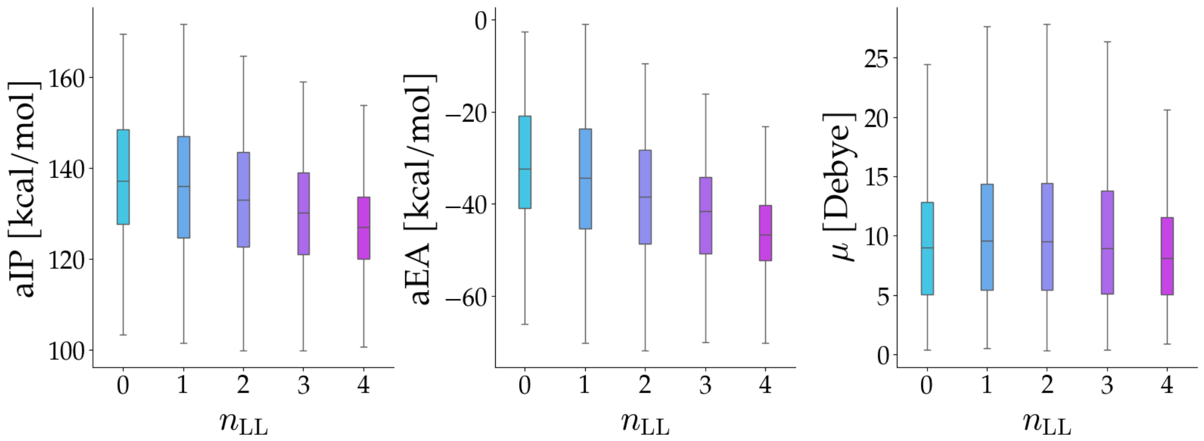

Figure S11: The effect of n_{LL} on A) aIP (in kcal/mol), B) aEA (in kcal/mol), and C) dipole moment μ (in Debye). For aIP and aEA, molecules with negative frequencies in their charged states were ignored. Also, only n_{rings}=6 isomers are included.

S5.3 Feature #3: B_i/B_o, N_i/N_o

The previous two features —n_{rings} and n_{LL}—are both scaffold-based features, which exhibit clear trends for aIP and aEA but are not effective for accurate analysis/prediction of μ. Yet, as we explained in the previous subsections, this is not surprising, as we expect that μ will be determined by the location of B and N within the scaffold.

Figure S12 presents the distributions of aIP, aEA, and μ as a function of the substitution site. When both N and B are in outer positions, both the aIP and aEA obtain lower values and a broader distribution. When both heteroatoms are in inner positions, the aIP and aEA values are higher, and the distribution narrows. Apart from the fact that the former subset is larger (more opportunities to be placed in an outer position than an inner one), the different property ranges can be linked to the formal charges of the heteroatoms in the neutral molecule. Heteroatoms in inner positions necessarily have a formal charge, which makes impedes their ability to stabilize charge.

For μ, we observe that there the values are higher when both heteroatoms are in outer positions than when they are in inner positions, and the mixed cases show a very similar distribution in an intermediate property range.

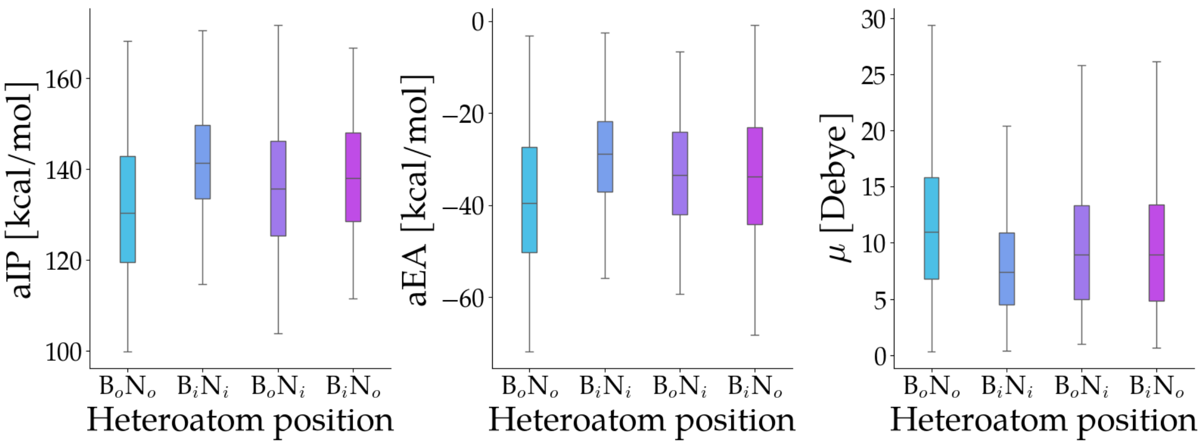

Figure S12: The effect of inner/outer positions on A) aIP (in kcal/mol), B) aEA (in kcal/mol), and C) μ (in Debye). Only n_{rings}=6 isomers are included.

S5.4 Feature #4: Shortest Path (n_{SP})

Having seen the influence of the substitution site in $(BN)_1$-PBHs on electronic properties, we next investigated the effect of their relative positions via the n_{SP} feature. Figure S13 shows the distributions of aIP, aEA, and μ versus n_{SP}. As n_{SP} increases, both aIP and aEA decrease. The rationalization for these trends is similar to what we propose in the main text for ΔE_{H-L} and E_{rel}. Namely, the effective polyene length in the $(BN)_1$-PBH is the conjugated path between B and N. As a result, the larger n_{SP} is, the the higher the HOMO energy will be and the lower the LUMO energy will be. According to Koopmans' Theorem, this then leads to a lower aIP and a more negative aEA. This trend was addressed in the main text and attributed to the different Clar sextets and quinoid-like structures that arise due to an odd/even n_{SP}.

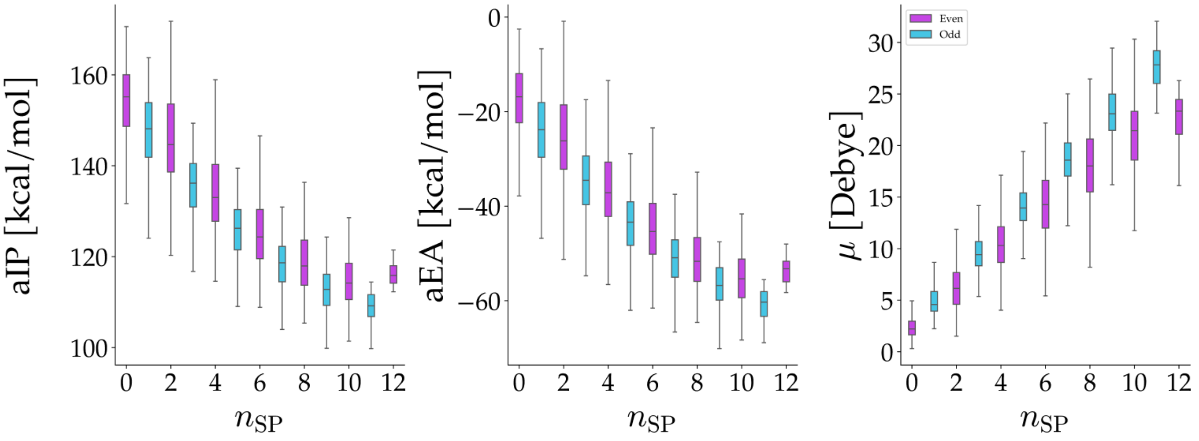

Figure S13: The effect of n_{SP}on A) aIP (in kcal/mol), B) aEA (in kcal/mol), and C) dipole moment μ (in Debye). For aIP and aEA, molecules with negative frequencies in their charged states were ignored. Also, only n_{rings}=6 isomers are included.

In contrast, μ increases as n_{SP} increases, as expected. This is because the value of μ is linearly correlated to the size of the charge separation (i.e., $\delta+$ and $\delta-$) and the distance between them. We also observe that $(BN)_1$-PBHs with an odd n_{SP} follow a slightly different linear trend than those with even n_{SP}, which may be due to the partial charges that are formed when allylic or vinylic substructures are formed (see main text).

Importantly, n_{SP} counts the number of carbons between B and N, not the geometric distance between them. Depending on the shape of the path between B and N, the same n_{SP} value can describe different geometric distances. Furthermore, the greater n_{SP} is, the more options for various routes. This is why we observe increasingly distributed values as n_{SP} increases.

To further investigate the influence of the shortest path on μ, we analyzed the deviation of μ (θ) from the N-to-B orientation in $(BN)_1$-PBHs (see Figure S14). The deviation (θ) is calculated as the dot-product between the N-to-B vector and the dipole vector. A high θ implies that the position of B and N has little influence on μ; a low θ implies that μ is determined by the position of the B and N. Figure S15 depicts the distribution of θ values across all $(BN)_1$-PBHs in COMPAS-4D. The maximal value of θ is 45°, with most molecules showing $\theta \leq 7°$. Additionally, we observe that θ decreases as n_{SP}increases, which indicates that heteroatoms play a larger role in dictating μ when they are farther apart. Because μ is influential in determining bulk properties (e.g., crystal packing orientation), this understanding of structure-property relationships may guide synthetic efforts.

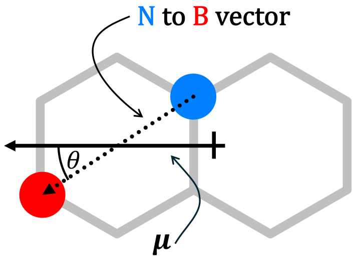

Figure S14: Schematic showing how dipole vector deviation (θ) from heteroatom orientation was calculated.

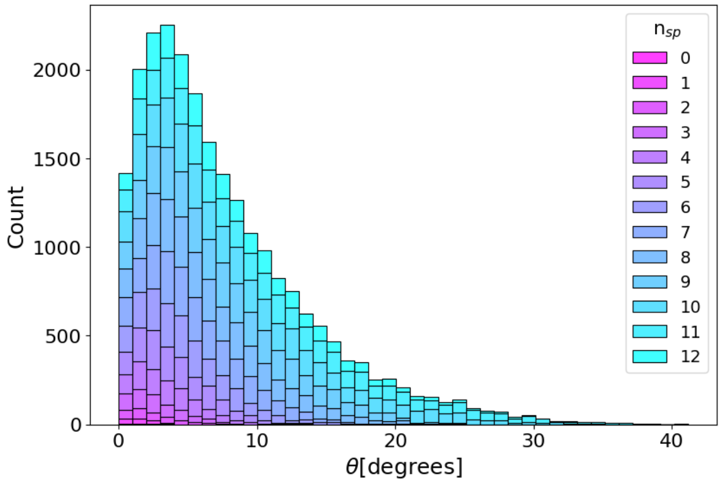

Figure S15: Stacked histogram of deviations of the dipole vector from the B to N orientation vector in COMPAS-4D classified based on n_{SP}.

S5.5 Feature #5: Number of disturbed rings n_{DR}

As described in the main text, the incorporation of B and N into a PBH disrupts not only the conjugated circuit but also the aromatic character of the molecule. The larger the number of disrupted rings, the greater the loss of aromaticity. This inherently affects the MO levels: the greater n_{DR} the higher HOMO will be and the lower LUMO will be. According to Koopmans' Theorem, this results in lower aIP and more negative aEA (exactly as is shown in Figure S16).

It is interesting to note that μ is also dependent on n_{DR}. We believe this observed effect is not due to a direct relationship between μ and aromaticity, but rather to the fact that a larger n_{DR} necessarily indicates a greater distance between B and N (as was seen in the analysis of n_{SP}).

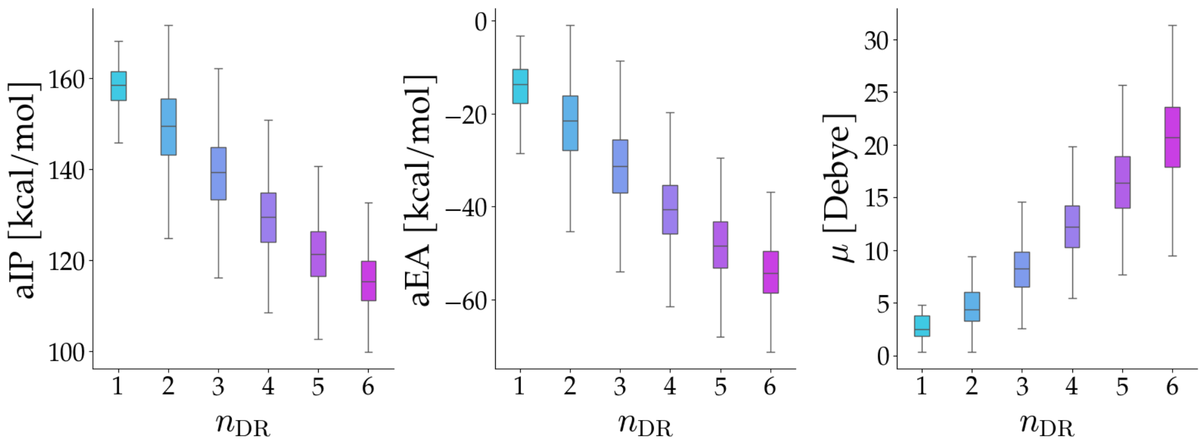

Figure S16: The effect of n_{DR} on A) aIP (in kcal/mol), B) aEA (in kcal/mol), and C) dipole moment μ (in Debye). Only n_{rings}=6 isomers are included.

S6 Regression analyses

In the main manuscript, we explored how well the Light-Gradient Boosting Method (LGBM) models[10] predict $\Delta E_{\mathrm{H-L}}$ and E_{rel} using only the aforementioned five features. In this section, we describe a comparison between various models and check their predictive performance for different electronic properties, including $\Delta E_{\mathrm{H-L}}$, E_{rel}, aIP, aEA, and μ. Finally, to confirm the importance of each feature used in our models, we performed ablation studies. All models were used out-of-the-box without any hyperparameter tuning. Most regression tasks, including data pre-processing, cross-validation, and regression analyses were performed by methods provided in scikit-learn unless explicitly mentioned.[11]

S6.1 Regression protocol

1. COMPAS-4D data were split into training (75%) and test set (25%).
2. We performed 5 cross-validation splits using ShuffleSplit on the training set with 25% of the training data used as the validation set and 75% used for training.
3. We then trained a final model on the entire training set.
4. This final model was then evaluated on the test set.
5. We then compared the cross-validation performance to the test data's performance via the Wilcoxon signed-rank test.

From the final step, we ensured that the final results and cross-validated scores were not statistically significant.

S6.2 Performance of different models on $\Delta E_{\mathrm{H-L}}$ and E_{rel}

Apart from LGBM, the three other models we explored were Random Forest (RF)[12] and CatBoost[13] (both radial tree-based models), and linear regression (LR). Figure S17 shows that, for $\Delta E_{\mathrm{H-L}}$, the radial tree-based models outperform LR and achieve comparable performance to LGBM. Cross-validated MAEs for RF, CatBoost, and LR were 0.27 ± 0.00, 0.26 ± 0.00, and 0.41 ± 0.00, respectively. Each model presented negligible uncertainty during their cross-validation when predicting $\Delta E_{\mathrm{H-L}}$. Figure S18 shows that, for E_{rel}, CatBoost performs better than RF while both radial tree-based models models outperform LR. Cross-validated mean absolute errors for RF, CatBoost, and LR were 4.25 ± 0.04, 4.18 ± 0.03, and 8.48 ± 0.07 respectively.

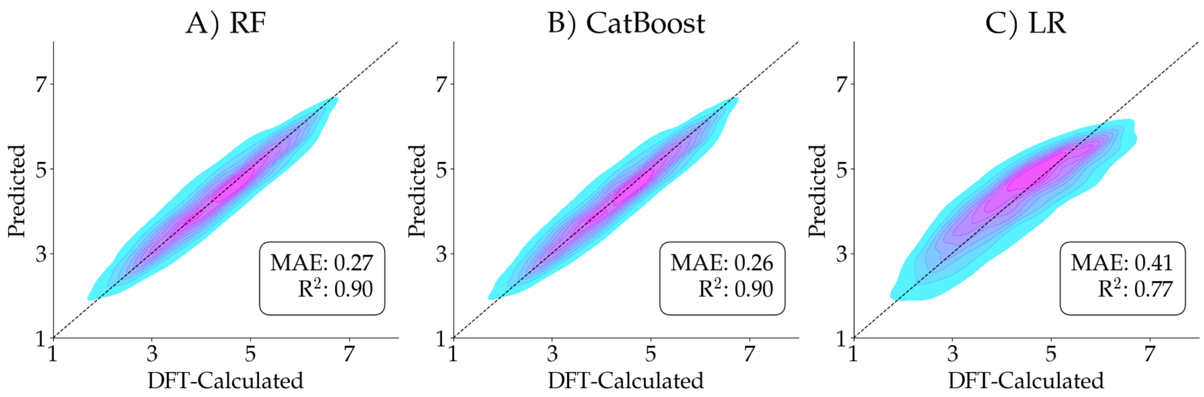

Figure S17: $\Delta E_{\mathrm{H-L}}$ as predicted by Random Forest Regression (RF), CatBoost Regression (CatBoost), and linear regression (LR). Also provided are the Mean absolute error (MAE), and coefficient of determination (R^2) for the final models. All values are in eV.

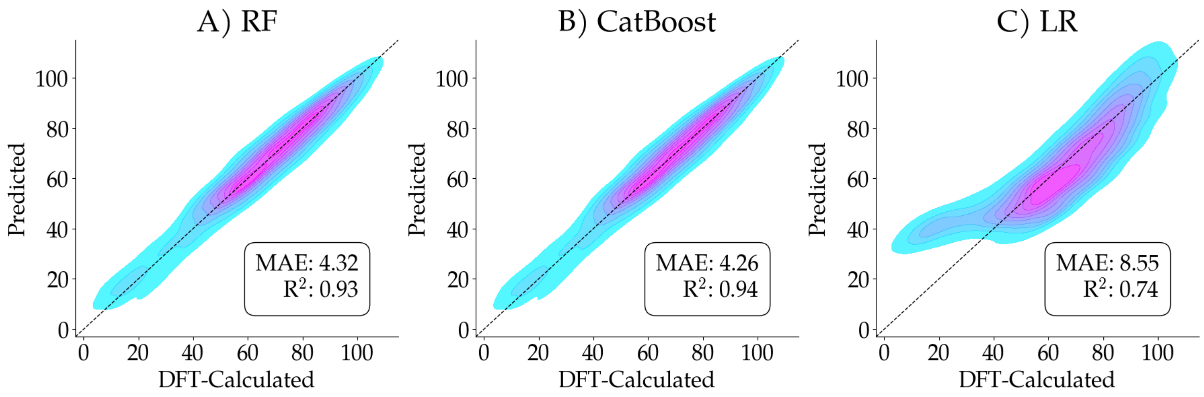

Figure S18: E_{rel} as predicted by Random Forest Regression (RF), CatBoost Regression (CatBoost), and linear regression (LR). Also provided are the Mean absolute error (MAE), and coefficient of determination (R^2) for the final models. All values are in kcal/mol.

S6.3 aIP, aEA, and μ

Next, we investigated all four models' performance for aIP, aEA, and μ. In all cases, the performance was evaluated on the 25% of the data reserved for testing. The predicted values are plotted against the DFT-calculated values. MAE and R^2 are noted on each plot. MAEs for aIP and aEA are in kcal/mol; MAE for μ is in Debye. All four models predict μ better than aIP and aEA. All radial tree-based models performed similarly and outperformed LR. The reasonable accuracies of diverse radial tree-based models on different electronic properties establish the robustness of our features at predicting electronic properties of $(BN)_1$-PBHs.

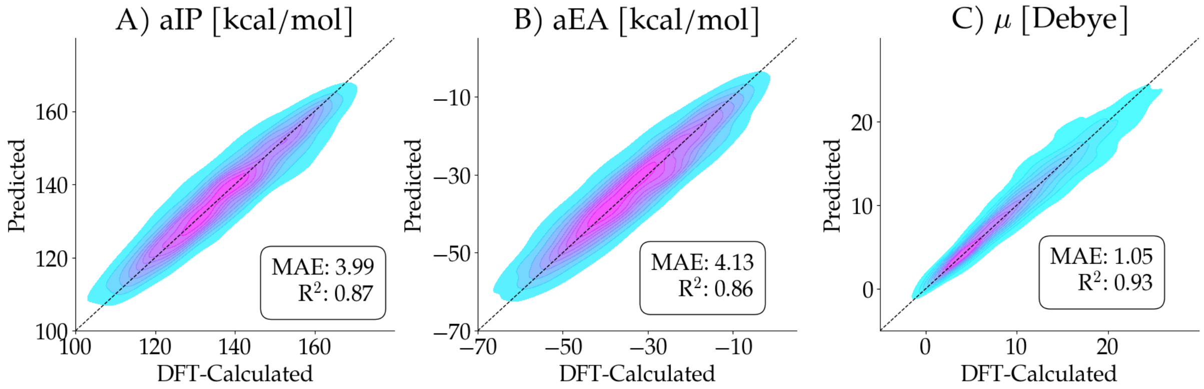

Figure S19: Performance of LGBM regressor models for prediction of A) aIP, B) aEA, and C) μ.

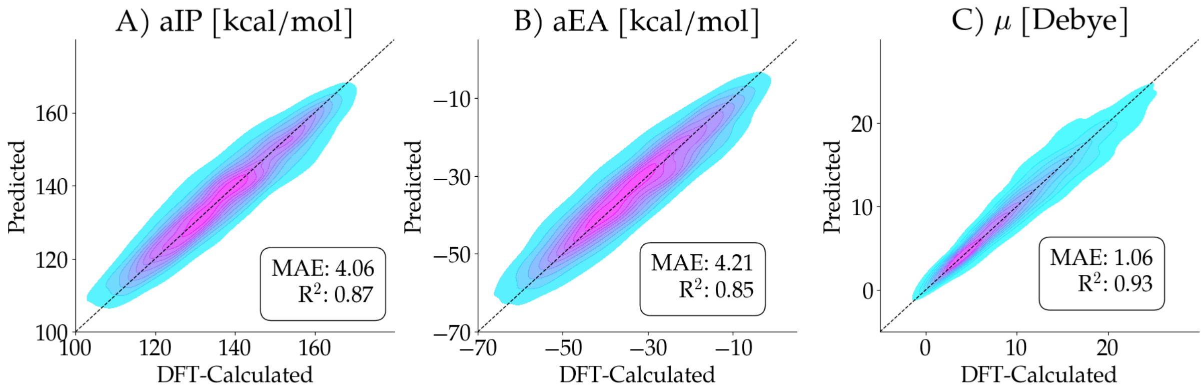

Figure S20: Performance of RF regressor models for prediction of A) aIP, B) aEA, and C) μ.

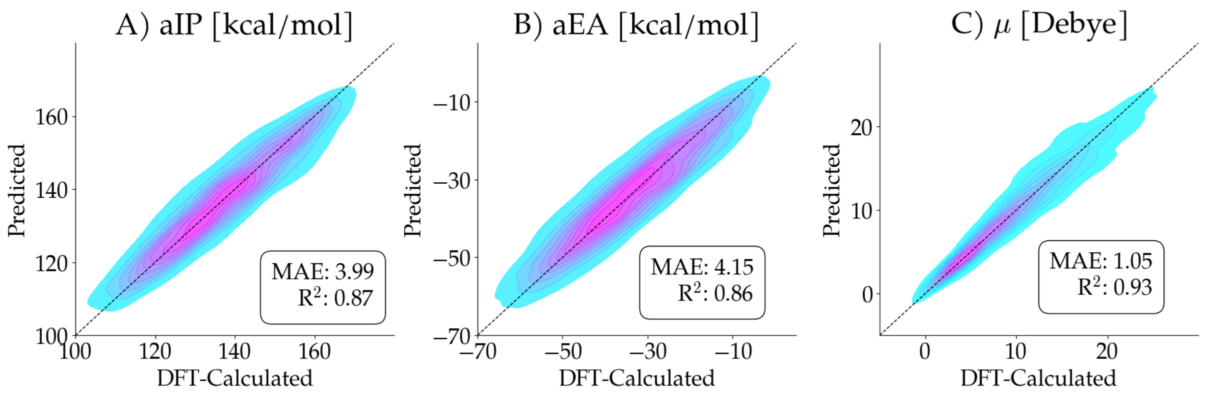

Figure S21: Performance of CatBoost regressor models for prediction of A) aIP, B) aEA, and C) μ.

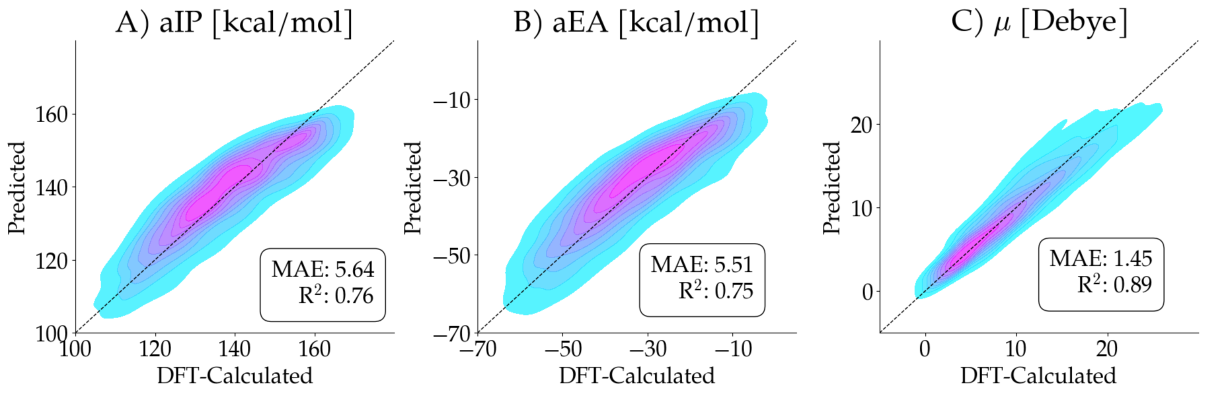

Figure S22: Performance of LR models for prediction of A) aIP, B) aEA, and C) μ on the test set (25% of data). MAE and R^2 are noted on each plot. MAEs for aIP and aEA are in kcal/mol; MAE for μ is in Debye.

S6.4 Ablation studies

To prove the usefulness of our engineered features, we performed leave-one-out ablation studies, iteratively removing one of the features from the input and evaluating the predictive performance of the resulting model.

Figure S23 compares the performance of the original model (trained using all features) in predicting $\Delta E_{\text{H-L}}$, to five new models, each one trained with one omitted feature. Interestingly, there was no noticeable loss in accuracy when we removed n_{rings}, despite the clear relevance of this feature that was demonstrated in the main text. The reason for this is that our dataset is predominantly composed of $(BN)_1$-PBHs with n_{rings}=6, which far outweigh any of the other size subsets. All other models show a reduction in performance when a feature is removed. In particular, n_{DR} stands out as the most dominant feature. This analysis demonstrates that all features are non-redundant and critical to the performance of the model.

For E_{rel}, we observe again that n_{rings} is seemingly not important, which we commented on above. Omission of each of the other features shows a clear reduction in model performance. In this case, the feature that appears to have the greatest effect is n_{SP}. Nonetheless, all of the features are necessary for the success of the prediction.

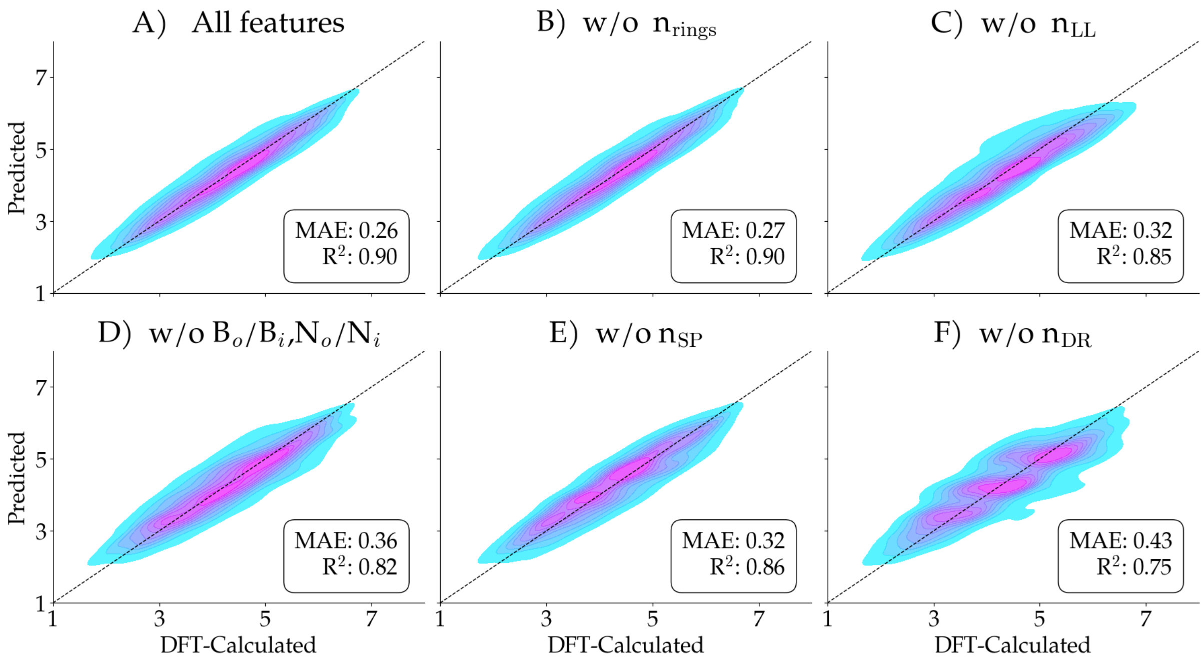

Figure S23: LGBM ablation study for $\Delta E_{\mathrm{H-L}}$.

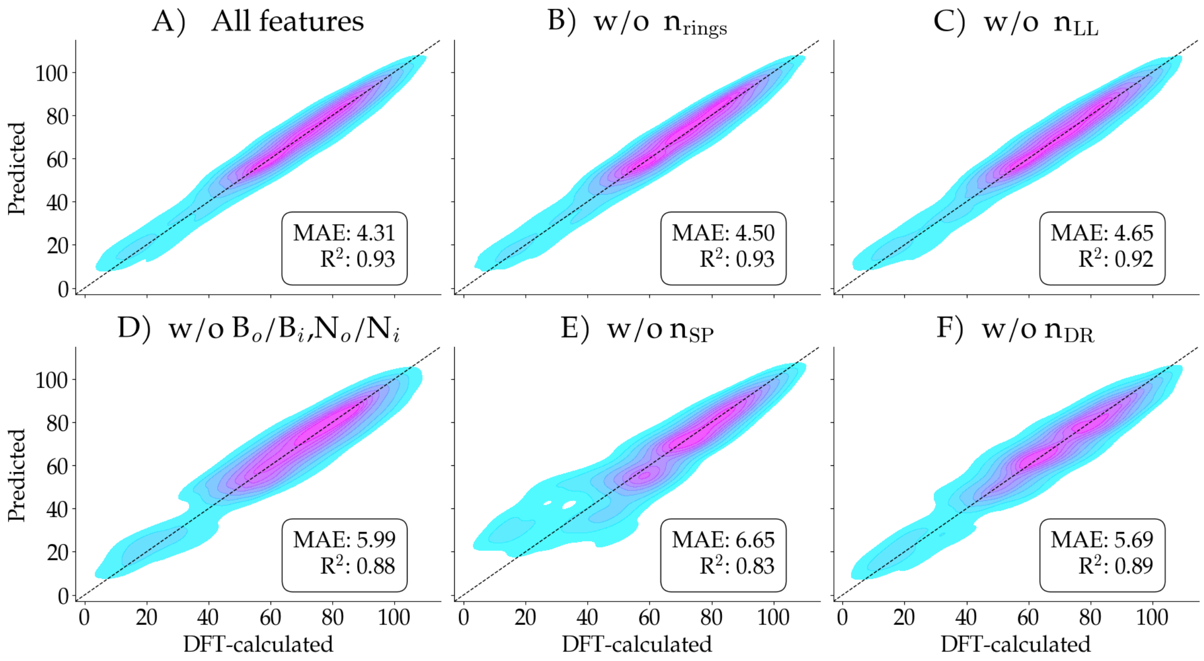

Figure S24: LGBM ablation study for E_{rel}.

S7 References

References

(1) Wahab, A.; Pfuderer, L.; Paenurk, E.; Gershoni-Poranne, R. *J. Chem. Inf. Model.* **2022**, *62*, 3704–3713.

(2) Mayo Yanes, E.; Chakraborty, S.; Gershoni-Poranne, R. *Sci Data* **2024**, *11*, 97.

(3) Markert, G.; Paenurk, E.; Gershoni-Poranne, R. *Chem. Eur. J.* **2021**, *27*, 6923–6935.

(4) Khaleel, F.; Chakraborty, S.; Gershoni-Poranne, R. *J. Phys. Org. Chem.* **2025**, *38*, e70012.

(5) Liu, Z.; Ishibashi, J. S.; Darrigan, C.; Dargelos, A.; Chrostowska, A.; Li, B.; Vasiliu, M.; Dixon, D. A.; Liu, S.-Y. *J. Am. Chem. Soc.* **2017**, *139*, 6082–6085.

(6) Ishibashi, J. S.; Marshall, J. L.; Maziere, A.; Lovinger, G. J.; Li, B.; Zakharov, L. N.; Dargelos, A.; Graciaa, A.; Chrostowska, A.; Liu, S.-Y. *J. Am. Chem. Soc.* **2014**, *136*, 15414–15421.

(7) Abengózar, A.; Sucunza, D.; García-García, P.; Sampedro, D.; Pérez-Redondo, A.; Vaquero, J. J. *J. Org. Chem.* **2019**, *84*, 7113–7122.

(8) Abengózar, A.; García-García, P.; Sucunza, D.; Frutos, L. M.; Castaño, O.; Sampedro, D.; Pérez-Redondo, A.; Vaquero, J. J. *Org. Lett.* **2017**, *19*, 3458–3461.

(9) Abengozar, A.; Garcia-Garcia, P.; Sucunza, D.; Sampedro, D.; Pérez-Redondo, A.; Vaquero, J. J. *Org. Lett.* **2019**, *21*, 2550–2554.

(10) Ke, G.; Meng, Q.; Finley, T.; Wang, T.; Chen, W.; Ma, W.; Ye, Q.; Liu, T.-Y. *NeurIPS* **2017**, *30.*

(11) Pedregosa, F. et al. *J. Mach. Learn. Res.* **2011**, *12*, 2825–2830.

(12) Breiman, L. *Machine learning* **2001**, *45*, 5–32.

(13) Prokhorenkova, L.; Gusev, G.; Vorobev, A.; Dorogush, A. V.; Gulin, A. *NeurIPS* **2018**, *31.*
